# Three-Dimensional Printing Resin-Based Dental Provisional Crowns and Bridges: Recent Progress in Properties, Applications, and Perspectives

**DOI:** 10.3390/ma18102202

**Published:** 2025-05-10

**Authors:** Xiaoxu Liang, Biao Yu, Yuan Dai, Yueyang Wang, Mingye Hu, Hai-Jing Zhong, Jingwei He

**Affiliations:** 1 School of Arts and Sciences, Guangzhou Maritime University, Guangzhou 510725, China; liangxxu@126.com; 2 School of Chemistry and Chemical Engineering, Lingnan Normal University, Zhanjiang 524048, China; y.biao@lingnan.edu.cn; 3 School of Health Preservation and Rehabilitation, Chengdu University of Traditional Chinese Medicine, Chengdu 611137, China; daiyuan@cdutcm.edu.cn; 4 Joint Key Laboratory of the Ministry of Education, Institute of Applied Physics and Materials Engineering, University of Macau, Avenida da Universidade, Taipa, Macau SAR, China; yc17813@um.edu.mo; 5 State Key Laboratory of Bioactive Molecules and Druggability Assessment, Jinan University, Guangzhou 510632, China; hmyyye712@163.com; 6 School of Materials Science and Engineering, South China University of Technology, Guangzhou 510641, China

**Keywords:** 3D printing, provisional crowns and bridges, digital dentistry, resin, properties, clinical application

## Abstract

Three-dimensional (3D) printing represents a pivotal technological advancement in dental prosthetics, fundamentally transforming the fabrication of provisional crowns and bridges through innovative vat photopolymerization methodologies, specifically stereolithography (SLA) and digital light processing (DLP). This comprehensive scholarly review critically examines the technological landscape of 3D-printed resin-based dental provisional crowns and bridges, systematically analyzing their material performance, clinical applications, and prospective developmental trajectories. Empirical investigations demonstrate that these advanced restorations exhibit remarkable mechanical characteristics, including flexural strength ranging from 60 to 90 MPa and fracture resistance of 1000–1200 N, consistently matching or surpassing traditional manufacturing techniques. The digital workflow introduces substantial procedural innovations, dramatically reducing fabrication time while simultaneously achieving superior marginal adaptation and internal architectural precision. Despite these significant technological advancements, critical challenges persist, encompassing material durability limitations, interlayer bonding strength inconsistencies, and the current paucity of longitudinal clinical evidence. Contemporary research initiatives are strategically focused on optimizing resin formulations through strategic filler incorporation, enhancing post-processing protocols, and addressing fundamental limitations in color stability and water sorption characteristics. Ultimately, this scholarly review aims to provide comprehensive insights that will inform evidence-based clinical practices and delineate future research trajectories in the dynamically evolving domain of digital dentistry, with the paramount objective of advancing patient outcomes through technological innovation and precision-driven methodological approaches.

## 1. Introduction

Provisional crowns and bridges are fundamental components in the treatment of fixed partial dentures. A well-fabricated provisional restoration serves multiple essential functions: protecting prepared teeth from thermal, chemical, and mechanical stimuli; maintaining periodontal health; stabilizing occlusal relationships; preserving spatial relationships; providing esthetic simulation of the final restoration; and allowing functional and esthetic evaluation prior to definitive treatment [1,2]. The clinical success of these restorations depends on their ability to meet stringent mechanical, biological, and esthetic requirements while maintaining dimensional accuracy [3,4,5,6].

Computer-aided design and computer-aided manufacturing (CAD/CAM) technologies have revolutionized prosthodontics through enhanced precision, standardization, and efficiency [6,7]. While subtractive manufacturing (milling) has dominated digital workflows for provisional restorations in recent years, additive manufacturing (3D printing) has emerged as a compelling alternative due to its material conservation, geometric freedom, and cost-effectiveness [5,8,9,10,11,12]. The clinical potential of 3D printing is particularly evident in fabricating provisional crowns and bridges, with single-unit restorations printable in approximately 20 min. This efficiency allows clinicians to prepare a tooth, scan it, initiate printing, and proceed with other treatments while the restoration is manufactured. Once complete, the crown can be immediately detached from supports and cemented, thereby streamlining clinical workflow without compromising restoration quality [13,14,15].

Among the various 3D printing technologies available for dental applications, vat photopolymerization methods—particularly stereolithography (SLA) and digital light processing (DLP)—have emerged as dominant approaches for fabricating provisional restorations [11,12]. These technologies utilize photosensitive liquid resins that polymerize upon exposure to specific wavelengths of light, enabling layer-by-layer construction of three-dimensional objects with high precision. Recent advancements in printer resolution, material formulation, and post-processing protocols have significantly enhanced the mechanical properties, biocompatibility, and esthetic characteristics of 3D-printed provisional restorations [16,17,18]. Despite these advancements, several challenges persist in the clinical application of 3D-printed provisional restorations. These include material degradation in the oral environment, inherent anisotropy due to layer-by-layer fabrication, the optimization of resin compositions, and the standardization of manufacturing parameters [16,17,18,19,20]. Additionally, the long-term clinical performance of these materials remains inadequately documented, necessitating a critical evaluation of their mechanical, physical, and biological properties relative to conventional alternatives [21].

This comprehensive review synthesizes evidence on 3D-printed provisional crowns and bridges, providing clinicians and researchers with a critical analysis of current capabilities, benefits, and limitations. We evaluate mechanical properties, physical properties, biocompatibility profiles, and clinical performance metrics to offer evidence-based guidance for material selection, manufacturing parameter optimization, and clinical implementation. As additive manufacturing transforms prosthodontic practice, understanding its potential and constraints becomes essential for enhancing patient care, developing improved materials, and establishing standardized protocols. By bridging the gap between laboratory findings and clinical application, this review facilitates the responsible integration of 3D printing technology into provisional crown and bridge workflows.

## 2. Three-Dimensional Printing Technology and Materials

### 2.1. Types of 3D Printing Technologies

**Figure 1:**
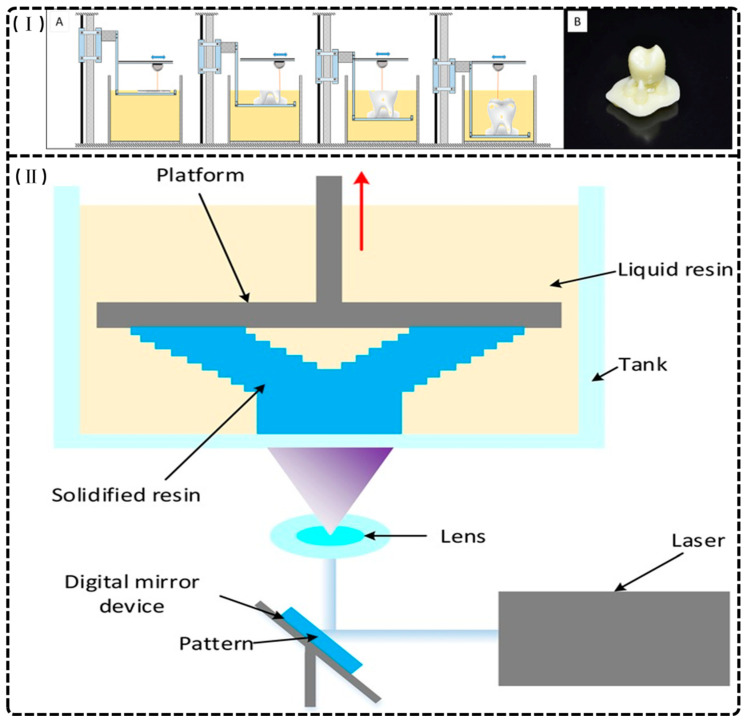
(**I**) Stereolithography printing. (**A**) The layer-by-layer printing process. (**B**) An example of a crown printed with stereolithography prior to the removal of supports and polishing. Reprinted with permission from [14]. (**II**) A schematic of the printer components and printing procedure of digital light projection (DLP), the red arrow represents the rising direction of the platform. Reprinted with permission from [22].

Dental additive manufacturing relies principally on SLA (as shown in Figure 1I) and DLP (as shown in Figure 1II), which cure photoreactive resins layer by layer via laser or projected light. These methods integrate directly with intraoral scans to produce highly accurate provisional crowns and bridges with minimal material waste and rapid turnaround [14,22].

#### 2.1.1. SLA

Stereolithography (SLA) represents a critical 3D printing technology in dentistry, distinguished by its exceptional dimensional accuracy and surface quality. The process employs layer-by-layer polymerization of liquid photopolymer resin via a focused ultraviolet laser [23,24] and operates in two configurations: top-down, where the build platform submerges in resin with incremental lowering, and bottom-up, where the laser cures resin as the platform descends [14,25]. The mechanical and physical properties of SLA-fabricated structures depend on layer thickness, orientation, polymerization parameters, and post-curing protocols [10,26]. This technology accommodates diverse photopolymer resins, including ceramic-filled variants, facilitating the production of study models, provisional restorations, surgical guides, and orthodontic appliances [14,23,24,27]. SLA enables the efficient creation of complex geometries and customized solutions while minimizing material waste, though potential dimensional inaccuracies may propagate from errors in earlier layers, affecting overall model precision. Despite this limitation, SLA’s extensive applications enhance prosthodontics, orthodontics, and surgical planning practices, ultimately improving patient outcomes through precise, tailored dental solutions [15,25,28].

#### 2.1.2. DLP

Digital light processing (DLP) is a 3D printing technology utilizing vat polymerization with a digital micromirror device (DMD) as its light source [14,22,29]. Unlike SLA, DLP projects patterned light that polymerizes entire resin layers simultaneously, with lateral resolution (10–50 μm) determined by micromirror density and vertical resolution governed by exposure-controlled curing depth [25,27,30,31,32,33]. DLP offers faster fabrication speeds than SLA, with its bottom-up configuration reducing oxygen inhibition during polymerization [34]. However, significant limitations include the requirement for low-viscosity resins, as empirical studies demonstrate that formulations exceeding 1500 cP produce structural defects including layer delamination and void formation [30,33]. The process necessitates post-fabrication UV curing and support removal, introducing workflow complexity and safety considerations [9]. While DLP produces satisfactory orthodontic casts and models with higher wear resistance than conventional resins [35], it neither consistently outperforms traditional dental stone for cast duplication nor matches traditional materials in mechanical durability for occlusal devices [36,37]. Despite these constraints, DLP remains valuable for precise, customized dental applications, offering an effective compromise between production efficiency and clinical performance.

### 2.2. Composition of Printable Provisional Resins

Advancements in AM have catalyzed the development of specialized polymeric resins for dental 3D printing applications, particularly for provisional restorations and denture teeth [11,38]. These materials typically comprise a controlled formulation of oligomers, monomers, photoinitiators, stabilizers, and pigments to achieve desired mechanical and optical properties [39,40,41,42,43]. Representative commercial formulations illustrate this compositional strategy: CLEAR FLGP04 incorporates methacrylate oligomer (75–95%), methacrylate monomer (25–50%), and diphenyl oxide (2,4,6-trimethylbenzoyl) phosphine (<1%) as a photoinitiator [44], while Everes Temporary (Sisma, Vicenza, Italy) utilizes aliphatic difunctional methacrylate (<50%), 2,2-ethylenedioxydiethyl dimethacrylate (<40%), aliphatic urethane acrylate (<20%), and phosphine oxide (<2.5%) to achieve application-specific performance characteristics [45].

**Figure 2:**
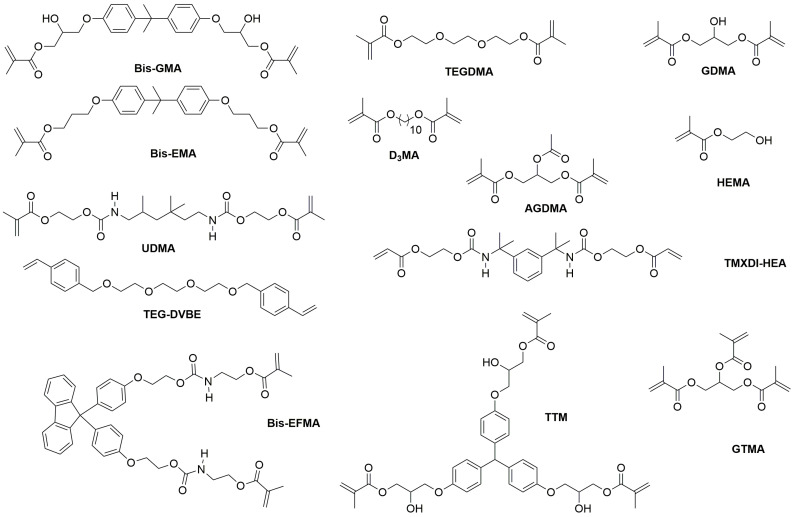
The chemical structure of monomers used in dental resin.

Photopolymerizable monomers—reactive molecules capable of forming polymeric networks—undergo radical or cationic polymerization via light-activated initiators, facilitating cross-linking critical for mechanical stability [9,46]. Contemporary dental resins combine high-molecular-weight base monomers (2,2-bis[4-(2-hydroxy-3-methacryloyloxypropoxy) phenyl] propane (BisGMA), ethoxylated BisGMA (Bis-EMA), and urethane dimethacrylate (UDMA)) with diluents (triethyleneglycol dimethacrylate (TEGDMA), decanediol dimethacrylate (D_3_MA), and 2-hydroxyethyl (HEMA)) to optimize viscosity and polymerization kinetics (Figure 2) [47,48]. These formulations exploit carbon–carbon double bonds to achieve rapid polymerization, yielding requisite mechanical strength and dimensional stability [30,41,49]. Though widely used since 1962, BisGMA (MW 512.6 g/mol, η = 1369 Pa·s) requires blending with low-viscosity TEGDMA (η = 0.05 Pa·s) to improve handling and degree of conversion [47,50]. Alternative monomers address specific limitations: Bis-EMA (η = 0.9 Pa·s) reduces shrinkage stress, glycerol dimethacrylate (GDMA), acetylated glycerol dimethacrylate (AGDMA) and glycerol trimethacrylate (GTMA) mitigate toxicity concerns, and BPA-free formulations (e.g., 9,9-Bis[4-((2-(2-methacryloyloxy)ethyl-carbamate)ethoxy)phenyl]fluorene (Bis-EFMA), trimethacrylate tris(4-hydroxyphenyl)methane triglycidyl methacrylate (TTM)) address biocompatibility issues [51,52,53]. Lin et al. demonstrated that optimized blends (80% Bis-EMA, 10% UDMA, 10% TEGDMA) achieve 0.051 mm accuracy with flexural strengths of 60–90 MPa [30], while novel urethane acrylics (urethane acrylate TMXDI-HEA) exhibit superior mechanical properties—40% higher modulus and 21% greater flexural strength than UDMA [54]. Despite these advancements, diluent-induced polymerization shrinkage has prompted investigation of alternatives like triethylene glycol divinylbenzyl ether TEG-DVBE, which enhances hydrolytic stability when combined with UDMA [55].

On other hand, bioresource derivative monomers present promising alternatives to synthetic counterparts in dental materials [56,57,58,59,60]. The niacin-derived antibacterial monomer 1,3-bis(methacryloyloxy)propyl-carbonyl-hexylpyridinium bromide (QANMA) demonstrates optimal performance at 10 wt% concentration, exhibiting significant antibacterial activity while maintaining flexural strength that meets ISO standards [56]. Similarly, methacrylate-functionalized betulin derivatives (M1Bet and M2Bet) synthesized via esterification reactions serve as effective antibacterial comonomers, partially or completely replacing Bis-GMA. Dental resins incorporating 10 wt% M2Bet in place of Bis-GMA exhibit comparable viscosity, higher light transmittance, an improved degree of conversion, and enhanced mechanical properties compared to conventional Bis-GMA/TEGDMA (50:50) formulations [57]. Magnolol derivative monomer-based materials further demonstrate advantages in conversion degree, reduced volume shrinkage, decreased surface roughness, and lower cytotoxicity compared to Bis-GMA-based alternatives [58]. Despite these promising results, the application of bioresource-derived monomers in 3D-printed dental crowns and bridges remains limited, indicating a significant area for future research and development.

Although 3D printing methodologies (SLA/DLP) minimize material waste compared to subtractive techniques, the limited availability of FDA-approved intraoral formulations (Table 1) [38,61], reliance on petrochemical feedstocks, and post-processing requirements (solvent cleaning, UV curing) still present ongoing challenges to widespread clinical implementation [62].

## 3. Properties and Performance

### 3.1. Mechanical Properties

Mechanical characterization constitutes the fundamental validation methodology for evaluating novel 3D-printed resins, enabling critical assessment against manufacturer specifications and conventional alternatives to determine clinical viability [17,38,63,64]. Standardized in vitro protocols quantify essential biomechanical parameters—including flexural strength, elastic modulus, and fracture resistance—that predict functional durability under masticatory loading [17,63]. Provisional crown and bridge materials must satisfy both mechanical requirements (ISO 4049: FS ≥ 80 MPa) [39,65,66,67,68] and esthetic demands for the chromatic and morphological replication of natural dentition [69,70]. This section systematically analyzes key mechanical properties across fabrication modalities to identify technological advancements and persistent challenges in achieving optimal biomechanical functionality, which are summarized in Table 2.

#### 3.1.1. Flexural Strength

Flexural strength (FS) represents a material’s resistance to deformation and fracture under bending loads, typically quantified via three-point bend testing [67]. Three-Dimensional printed provisional resins consistently demonstrate superior or comparable FS relative to conventional and milled alternatives [17,71,72,73]. Park et al. reported significantly higher FS values for DLP (1189 N) and SLA (1323 N) technologies compared to conventional methods (543 N), with no significant differences between the two printing technologies [72]. Similarly, Pantea et al. observed that commercial 3D-printed resins (NextDent C&B MFH: 141 ± 17 MPa; HARZ Labs Dental Sand: 143 ± 15 MPa) exhibited significantly higher bending strength than conventionally fabricated specimens (88 ± 10 MPa and 76 ± 7 MPa). This enhancement is attributed to superior material homogeneity achieved through additive manufacturing, suggesting 3D-printed provisional restorations represent viable alternatives to conventional fabrication methods [62,71].

#### 3.1.2. Elastic Modulus

Elastic modulus quantifies material stiffness under elastic deformation, typically determined as the slope of the stress–strain curve in the elastic region (0.05–0.25% strain). In dental materials research, this property is commonly measured via three-point bending tests according to ISO 4049 standards, yielding flexural modulus values. When direct tensile testing is employed, Young’s modulus is similarly calculated from the initial linear portion of the tensile stress–strain curve within the same strain range [15].

The elastic modulus of 3D-printed provisional materials is influenced by printing parameters, degree of conversion (DC), and material composition [15,17,62,72,74]. Comparative analyses yield inconsistent results across material classes. Pantea et al. documented significantly higher elastic modulus in MMA-based 3D-printed resins (6402 ± 69 MPa for 3DCS; 6329 ± 79 MPa for 3DOS) compared to conventional alternatives (4124 ± 333 MPa for CAP; 4022 ± 1167 MPa for CHP) [62]. Conversely, Simoneti et al. found lower values in MMA-based printed resins (513.3 MPa) versus conventional bis-acrylic materials (997.3 MPa), potentially reflecting differences in cross-linking density or molecular arrangement [15,75].

Manufacturing parameters significantly influence mechanical properties, with Tahayeri et al. demonstrating superior accuracy and mechanical integrity in specimens printed at 90° orientation [15]. Material composition further modulates performance, as evidenced by Ellakany et al., who reported higher elastic modulus in unfilled DLP AS resin (951.13 ± 68.61 MPa) compared to microfilled SLA ND resin (805.47 ± 190.37 MPa). This differential was attributed to the denser structure formed by photopolymerized methacrylate in DLP, contrasting with the increased flexibility introduced by microfillers in SLA materials. Additionally, simultaneous layer polymerization in DLP technology produces more uniform internal structures compared to the sequential laser curing in SLA, potentially contributing to enhanced elastic modulus (ISO 10477-2020 standard) [76]. Tasin et al. also reported that composite-based 3D-printed provisional resins outperform conventional PMMA, CAD/CAM PMMA, and bis-acrylic resins [74]. From a clinical perspective, provisional materials should balance rigidity and resilience, ideally approximating dentin’s stiffness (≈18–25 GPa) rather than enamel’s significantly higher modulus (≈70–96 GPa). Although current provisional resins exhibit lower moduli (1–3 GPa), this relative compliance can advantageously absorb occlusal forces and reduce stress transfer to prepared teeth or implants. However, insufficient stiffness may lead to excessive deformation under load, compromising marginal fit, promoting microleakage, and risking restoration failure—limitations requiring further material optimization [99].

#### 3.1.3. Fracture Strength

Fracture strength represents the maximum force a material can withstand before catastrophic failure, providing critical insights into provisional restoration durability under functional loading [77]. Testing protocols typically involve cementing fabricated crowns onto standardized dies, followed by and mechanical loading to simulate clinical failure modes under masticatory loads [42,93].

Research reveals inconsistent outcomes regarding the comparative fracture resistance of 3D-printed provisional restorations [15,77,78]. Alam et al. reported superior performance of 3D-printed provisional crowns (1243.18 N) compared to CAD/CAM milled (960.84 N) and conventional (558.85 N) alternatives [78]. Similarly, Tahayeri et al. demonstrated comparable or superior peak stress values for printed specimens relative to conventional materials, indicating adequate fracture strength for clinical applications [15].

Contrasting findings have been reported by other investigators. Martín-Ortega et al. documented lower fracture resistance in 3D-printed crowns (anterior: 636.5 N; posterior: 321.3 N) compared to milled counterparts (anterior: 988.4 N; posterior: 423.8 N), suggesting potential clinical limitations despite values exceeding physiological masticatory forces [79]. This discrepancy has been attributed to manufacturing variables including layering technique, build orientation, and polymerization characteristics [79,80]. Abad-Coronel et al. demonstrated material-specific differences, with milled PMMA crowns (1427.9 N) exhibiting higher fracture resistance than 3D-printed hybrid materials (3DPPa: 1231.0 N) and polymers (3DPPb: 1029.9 N), though all materials demonstrated clinically acceptable performance [81]. These conflicting results highlight the need for standardized testing protocols for optimizing the mechanical performance of 3D-printed provisional restorations [49,79,80].

#### 3.1.4. Microhardness

Microhardness constitutes a critical determinant of mechanical integrity in additively manufactured dental restorations, correlating directly with polymerization efficacy and flexural properties [7,82,83]. This parameter quantifies resistance to localized deformation under concentrated loading, predicting performance under masticatory forces while serving as an indirect measure of polymerization degree [73,75]. Standardized testing employs Vickers microhardness or Knoop hardness methodologies to generate comparative metrics across fabrication techniques and material compositions. Sufficient microhardness ensures structural integrity and clinical durability, whereas inadequate values may lead to premature wear, compromised marginal adaptation, and diminished performance under physiological loading [73,82].

Comparative analyses of microhardness across manufacturing methodologies yield material-specific outcomes. Revilla-León et al. documented significantly higher Knoop hardness in additively manufactured MMA-based interim resins (13.45 ± 2.93 KHN) compared to conventional bis-acrylic alternatives (4.92 ± 0.36 KHN) [82]. Similarly, Al-Qahtani et al. reported superior Vickers hardness in 3D-printed specimens (25.16 VHN) relative to CAD/CAM alternatives (22.07 VHN), though these printed materials simultaneously demonstrated higher surface roughness (5.77 μm) [73]. However, manufacturing parameters significantly influence these properties, as evidenced by Simoneti et al., who observed inferior hardness and consequent fatigue resistance in horizontally printed SLA Gray resin compared to selective laser sintering and conventional specimens—findings potentially attributable to suboptimal printing orientation rather than inherent material limitations [75]. These investigations collectively suggest that additively manufactured provisional restorations, when optimally processed, offer mechanical properties comparable or superior to traditional fabrication methods.

Multiple processing variables modulate the microhardness of additively manufactured dental resins, with post-polymerization protocols exerting particularly significant influence. Extended post-curing durations (15–20 min) substantially enhance hardness throughout the material thickness, though curing periods exceeding 60 min yield diminishing returns [7,83,100]. Thermal conditions during post-processing similarly affect mechanical outcomes, with elevated temperatures significantly improving Vickers hardness and degree of conversion, though excessive thermal exposure risks compromising esthetic properties through color alteration [7,84]. Material composition further influences hardness profiles, with photoinitiator formulation, filler content, and monomer chemistry determining both absolute hardness values and their distribution throughout the restoration [17,75,76]. Notably, hardness heterogeneity with depth represents a consistent challenge, as limited light penetration during curing creates differential polymerization gradients that result in harder surface layers compared to deeper regions—a consideration particularly relevant for thicker restorations [83,84]. These findings underscore the necessity of optimizing post-polymerization protocols and material selection to balance mechanical performance with esthetic requirements in additively manufactured provisional restorations.

In brief, hardness is a crucial property of 3D-printed resins used in dental applications, influencing mechanical strength, durability, and esthetics. Optimizing hardness through precise control of the polymerization process, post-curing duration, and material composition is essential for producing effective, durable, and visually acceptable dental restorations.

#### 3.1.5. Wear Resistance and Frictional Properties

The wear resistance of 3D-printed resin materials is critical for dental applications, particularly in provisional restorations. Wear resistance refers to a material’s ability to resist surface degradation caused by friction, abrasion, or mechanical interactions with opposing materials. It is typically assessed by measuring the volume or depth of material loss under simulated or real-life conditions, such as chewing cycles in dental studies. High wear resistance signifies minimal material loss and greater durability, enabling these materials to endure the forces associated with oral function. Studies have shown that 3D-printed resins exhibit wear resistance comparable to conventional milled and self-cured resins. Understanding wear patterns is essential for selecting appropriate materials, and addressing challenges related to interlayer bonding can further improve their clinical performance. However, additional research is needed to fully evaluate their physical properties and broaden their applications in dental practice [35,40,69,85].

Comparative analyses of wear characteristics across fabrication methodologies reveal material-specific differences in performance. Aldahian et al. documented significantly lower volumetric surface loss in 3D-printed specimens (10.81 ± 2.00 mm^3^) following cyclic loading compared to conventional (17.79 ± 2.78 mm^3^) and CAD/CAM alternatives (13.68 ± 1.7 mm^3^), suggesting enhanced wear resistance in additively manufactured materials [86]. Park et al. similarly observed comparable wear resistance between DLP-printed resins and milled or self-cured alternatives when subjected to 30,000 thermo-cycled chewing cycles against zirconia and metal antagonists, with no significant differences in volumetric loss or maximum depth measurements [40,88]. Complementary findings from Cha et al. demonstrated equivalent wear resistance between additively manufactured denture teeth resins and conventional alternatives under standardized stress conditions [25]. However, material-specific variations exist, as evidenced by Kessler et al., who reported that only 3Delta temp among tested additively manufactured resins exhibited wear performance comparable to conventional composite materials during three-body wear testing over 20,000 cycles [87].

Distinctive wear patterns observed in additively manufactured specimens reveal potential limitations in interlayer bonding strength. Park et al. documented characteristic crack propagation and interlayer delamination in 3D-printed materials opposed to metal antagonists, highlighting the anisotropic nature of these materials with stronger intralayer than interlayer bonds [40,88]. This structural vulnerability presents a significant challenge for clinical implementation, particularly in high-stress applications. Optimization strategies should address these mechanical anisotropies through refined post-processing protocols and material formulation modifications. Comprehensive characterization of additional mechanical properties—including flexural, compressive, tensile, shear, and fatigue parameters—remains necessary to fully validate the clinical potential of additively manufactured provisional materials across diverse applications [40,86,88,101]. Despite these challenges, current evidence supports the viability of 3D-printed resins for provisional restorations, provided appropriate material selection and manufacturing parameters are implemented.

Frictional properties in dental materials science integrate mechanical performance, biocompatibility, and clinical longevity, quantified through the coefficient of friction (COF)—the ratio of frictional to normal force at material interfaces. This parameter directly relates to Archard’s wear equation and material degradation mechanisms. In additive manufacturing, tribological properties are modulated by printing parameters (layer thickness, curing time, light intensity, and orientation), with finer layers and optimal curing enhancing performance through improved surface topography and cross-linking density [102,103].

Recent innovations demonstrate promising advancements, particularly nanodiamond reinforcement (0.1 wt%) in 3D-printed PMMA resins, which significantly reduced friction against stainless steel antagonists and improved wear resistance by 50–60% against both stainless steel and titanium. These materials also exhibited increased hardness (17%) and reduced bacterial adhesion (48%) without compromising printability, though in vivo validation remains necessary [104].

The stability of these properties under physiological conditions represents another critical consideration. Studies of hydrolytic aging (37 °C, 6 months in artificial saliva) reveal generally increased friction coefficients and decreased wear resistance post-aging, with significant material-specific variability. Despite these challenges, additively manufactured materials demonstrate superior mechanical properties compared to conventional alternatives, offering valuable insights for applications in temporary dental restorations where tribological performance directly influences clinical outcomes and patient comfort [105].

In summary, the evidence across multiple studies indicates that 3D-printed provisional restorations exhibit comparable or superior mechanical properties to conventional and CAD/CAM alternatives. Flexural strength values typically range from 60 to 90 MPa for well-formulated materials, with fracture resistance values of 1000–1200 N. The mechanical performance appears to be significantly influenced by post-processing protocols, printing parameters, and material composition. These findings suggest that 3D-printed resins can meet the mechanical requirements for provisional restorations.

### 3.2. Mechanical Property Enhancement

The mechanical properties of 3D-printed materials are influenced by factors such as filler incorporation [45], printing orientation [26], layer thickness [63], cleaning method and post-curing processes [85,89].

#### 3.2.1. Filler

The incorporation of fillers, such as glass silica and zirconia nanoparticles, has shown great potential in improving the mechanical, physical, and biological properties of the 3D-printed dental resins. This is particularly important for temporary crowns that must withstand functional stresses until permanent restorations are placed [15,42,45,88,89,90]. Zirconia nanoparticles significantly reinforce the resin matrix, improving flexural strength and resistance to cracking and deformation under load [106,107,108]. Glass fillers also improve flexural strength, although their effectiveness depends on the correct concentration and uniform distribution. In addition, fused silica nanoparticles increase hardness and wear resistance by forming a denser, more compact structure, while zirconia nanoparticles further reduce the risk of wear, chipping and fracture over time [108,109]. The uniform dispersion of nanoparticles in the resin matrix also inhibits crack propagation through “dispersion strengthening”, improving the material’s resistance to stress and reducing the risk of fracture. Additionally, the modified resins demonstrate high biocompatibility, addressing concerns about cytotoxicity associated with traditional dental resins [45,88,110,111].

**Figure 3:**
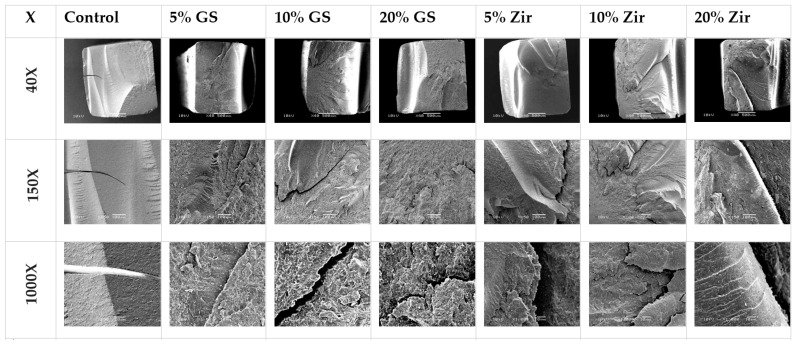
Representative SEM images (magnification 40×, 150×, and 1000×) of the fracture surface of tested groups: control, glass silica 5 wt% (GS 5%), glass silica 10 wt% (GS 10%), glass silica 20 wt% (GS 20%), zirconia 5 wt% (Zir 5%), zirconia 10 wt% (Zir 10%), and zirconia 20 wt% (Zir 20%) [45].

Filler content optimization critically influences mechanical performance, with Alshamrani et al. demonstrating that 5% glass and 10–20% zirconia fillers achieve peak flexural strength (134 ± 12 MPa) and Vickers hardness (82 ± 5 HV). However, exceeding the 20% threshold for glass fillers induces detrimental agglomeration and microstructural defects (as evidenced by SEM analysis (Figure 3)) which propagate stress concentration points that reduce fracture toughness by 18–22%. This nonlinear relationship between filler loading and material integrity establishes a critical optimization window for additive manufacturing parameters [45]. Similarly, KeßLer et al. systematically compared filler-dependent mechanical behavior in three 3D-printed resin composites, revealing critical compositional influences. 3Delta temp’s inorganic filler loading (50 wt%/30 vol%) was the highest among tested materials, contrasted with NextDent C&B and Free Print Temp’s unspecified (presumably lower) filler fractions. This compositional variance directly correlated with performance outcomes: 3Delta temp demonstrated quantitatively superior flexural strength (Δ + 27%) and modulus (Δ + 34%) compared to unfilled alternatives, consistent with established filler-reinforcement mechanisms. The findings underscore inorganic filler density as a key determinant of biomechanical efficacy in provisional restorative materials [90].

However, the incorporation of fillers in 3D printing resins also presents several challenges that significantly influence material performance [85]. During the printing process, suspended filler particles must maintain stable dispersion without sedimentation or agglomeration [112]. When particle clustering occurs, the increased inter-particle spacing compromises the protective function of fillers against abrasive forces on the resin matrix [113,114]. The mechanical properties of filled resins are largely dependent on particle size distribution and the efficiency of chemical coupling between fillers and the resin matrix [115]. Additionally, the filler–matrix interface has been identified as a potential initiation site for fatigue cracks. Observations of crack formation in filled resin restorations after simulated masticatory loading suggest that inadequate filler–matrix bonding may compromise the material’s structural integrity [85,116].

In short, the incorporation of these nanoparticles significantly enhances the mechanical properties, making the materials suitable for clinical applications such as temporary crowns that endure chewing stresses. These findings contribute to the advancement of additive manufacturing in dentistry, suggesting potential for broader applications and innovations in dental materials. Future research is needed to assess the long-term clinical performance and optimize formulations, which could further enhance the utility of these materials in restorative dentistry. Altogether, this work lays the groundwork for transformative advancements in dental materials and practices [45,90].

#### 3.2.2. Printing Parameters

The mechanical properties of 3D-printed provisional restorative resins are significantly influenced by manufacturing parameters, with layer thickness and printing orientation serving as critical determinants of structural integrity and performance characteristics. Layer thickness significantly influences the mechanical properties of 3D-printed provisional restorations through its complex interaction with polymerization dynamics. This parameter directly affects the degree of conversion and cross-linking density between successive layers, determining the material’s overall structural integrity through the interplay of light penetration depth, oxygen inhibition at interfaces, and post-curing effectiveness [63,117,118]. The optimization of layer thickness requires balancing print resolution, manufacturing efficiency, and mechanical consistency [80,85,91]. Alshamrani et al. demonstrated this relationship in their analysis of EVERES TEMPORARY resin printed at varying thicknesses (25, 50, and 100 μm), finding that 100 μm layers exhibited superior flexural strength (94.60 MPa) while 50 μm layers achieved maximum Vickers hardness (17.95 VHN), though these findings were limited to a single resin type and DLP technology [63,92].

**Figure 4:**
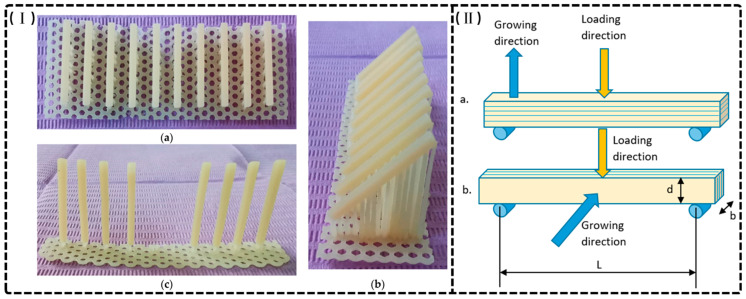
(**I**) The printed specimens made of DETAX on three different directions: 0, 45, and 90 degrees. (**a**) 0°; (**b**) 45°; (**c**) 90°. (**II**) Loading directions (**a**) perpendicular to growing direction; (**b**) parallel to growing direction. b—specimen thickness, d—specimen depth, L—span between supports [43].

The anisotropic nature of additive manufacturing significantly impacts the mechanical behavior of 3D-printed provisional restorations, with printing orientation critically influencing FS and modulus [4,43,86,89,90,93,94,101]. Turksayar et al. established that specimens printed at 0° and 30° orientations demonstrated fracture strengths comparable to conventional milled PMMA, while 90° specimens exhibited significantly compromised mechanical properties [93]. Derban et al. further elucidated orientation effects using SLA-fabricated NEXTDENT and DETAX resins (as shown in Figure 4), finding that parallel loading enhanced mechanical performance (Young’s modulus: +18–20%; flexural strength: +4.6–26.5%), with 90° orientation yielding optimal dimensional accuracy despite material-specific variations in brittleness and angular sensitivity [43]. The mechanical basis for these differences lies in interlayer adhesion quality: horizontally printed specimens demonstrate superior resistance to tensile and compressive forces, while vertically printed structures exhibit vulnerability to delamination along layer interfaces, exacerbated by manufacturing defects that function as stress concentrators [24,90,119,120]. KeßLer et al. confirmed that horizontal orientations consistently yield superior mechanical properties, underscoring orientation optimization as a critical consideration for achieving clinical durability in 3D-printed provisional restorations [90].

Overall, these findings provide valuable insights for clinical applications in prosthodontics by elucidating the relationship between fabrication parameters and mechanical performance.

#### 3.2.3. Cleaning Methods

In contrast to subtractive manufacturing, 3D printing resins require a distinct post-processing workflow comprising three main steps: surface cleaning to remove excess resin, support structure removal, and post-polymerization [19,85]. The cleaning focuses on eliminating residual uncured resin, typically achieved by immersing the object in an ultrasonic bath containing an organic solvent, such as isopropanol. Alternatively, centrifugal force has been suggested as an effective method for extracting uncured monomers. These processes are essential for ensuring the precision and mechanical integrity of the final product [19,85,121]. For instance, Mayer et al. investigated the effect of different cleaning methods (immersion in isopropanol (ISO), Yellow Magic 7 (YEL), and mechanical cleaning by centrifugal force (CEN)) on the fracture load of three-unit fixed dental prostheses (FDPs) made from different 3D printing resin-based materials. The specimens cleaned with ISO had lower fracture load values compared to those cleaned with CEN or YEL. This suggests that isopropanol has a detrimental effect on the mechanical properties of the resin. The proposed mechanism behind this negative effect is that isopropanol can dissolve the polymer matrix of the resin, leading to a disruption in the integrity of the material. The solvent is likely to interact with the polymer chains, causing them to swell or break apart, compromising the overall strength and durability of the printed prostheses. In contrast, the other cleaning methods (CEN and YEL) showed no negative effect on fracture load. This suggests that these methods may be more effective in maintaining the mechanical strength of 3D-printed dental restorations. The results highlight the importance of selecting appropriate cleaning methods to optimize the performance and longevity of 3D-printed dental restorations, as improper cleaning can lead to reduced mechanical properties and potential failure in clinical applications [85].

#### 3.2.4. Post-Polymerization

Three-dimensional printing of provisional dental restorations produces “green state” materials that remain partially polymerized following initial fabrication [96,97,122,123]. Post-polymerization represents a critical processing step that significantly enhances mechanical properties and biocompatibility through continued cross-linking of polymer chains. This process typically employs ultraviolet (UV) or near-UV light exposure, often combined with elevated temperatures, to facilitate increased monomer conversion beyond what is achieved during initial printing [15,122]. The resultant enhancement of polymer network density manifests as improved flexural strength, modulus, and hardness while simultaneously reducing residual monomer content, thereby optimizing both mechanical performance and biocompatibility of the printed restoration [123,124,125].

Temperature parameters significantly influence post-polymerization efficacy, as demonstrated by Chen et al., who established that exposure at 60 °C for 90 min produced optimal improvements in flexural properties. This enhancement occurs through thermally induced increases in molecular mobility and reaction kinetics that accelerate cross-linking reactions within the polymer matrix [96]. However, excessive thermal exposure introduces the risk of photodegradation through oxidative mechanisms, potentially compromising mechanical integrity rather than enhancing it [96,112,122]. These findings highlight the critical importance of precise temperature control during post-processing protocols to maximize mechanical performance while avoiding thermal degradation.

The duration of post-polymerization treatment similarly affects restoration quality, with Crispim et al. demonstrating that extended exposure time correlates with an increased degree of conversion (DC) and enhanced mechanical performance. Their investigation established that 30 min post-polymerization yielded optimal fracture resistance (844.30 N) in provisional crowns [97]. Conversely, inadequate post-polymerization results in inhomogeneous curing with trapped unreacted monomers, introducing dimensional instability through continued polymerization shrinkage during clinical service. Overall, this research underscores the necessity of optimizing both thermal and temporal parameters during post-processing to balance maximal polymerization against potential material degradation, thereby ensuring dimensional accuracy, mechanical integrity, and the clinical performance of 3D-printed provisional restorations.

#### 3.2.5. Aging Treatment

Environmental factors—particularly moisture and temperature fluctuations—significantly influence the mechanical properties of 3D-printed provisional restorations during clinical service [26,41,89]. Water sorption induces polymer matrix plasticization, causing chemical and physical changes that compromise material integrity through unreacted monomer release and by-product formation [26,126]. These degradative processes potentially affect structural stability and mechanical performance, necessitating investigation of artificial aging protocols to predict long-term clinical behavior and evaluate durability under simulated intraoral conditions [89,97,127].

Research examining aging effects on 3D-printed provisional materials demonstrates variable outcomes. Crispim et al. observed that water immersion (37 °C, 90 days) increased the degree of conversion (DC) of printed provisional crowns, suggesting enhanced polymerization of residual monomers, yet simultaneously reduced fracture resistance [97]. Similarly, Scherer et al. reported significant FS deterioration in NextDent C&B MFH specimens after thermocycling (6000 cycles), with values decreasing from 289.77 MPa to 207.17 MPa [92]. Conversely, Britto et al. demonstrated improved mechanical performance after long-term water immersion (six months, 37 °C), with 3D-printed polymer exhibiting increased flexural strength (48.5 MPa to 76.5 MPa) compared to heat-cured acrylic resin (63.7 MPa) and bis-acryl composite resin (12.4 MPa), though the mechanisms underlying this enhancement were not fully characterized [39].

Material-specific limitations exist regarding aging resistance. Henderson et al. established that 3D-printed bis-acryl resin interim prostheses exhibited inferior mechanical properties compared to milled PMMA and chairside auto-polymerizing bis-acryl for fixed dental prostheses. The 3D-printed material showed significant deterioration after extended humid storage, with mean failure loads decreasing from 520 N (1 day) to 363 N (30 days), while chairside auto-polymerizing bis-acryl maintained consistent strength throughout the testing period [49]. These findings highlight the differential susceptibility of various 3D-printed materials to hydrolytic degradation and underscore the need for material-specific optimization to enhance clinical longevity of 3D-printed provisional restorations.

In conclusion, 3D-printed provisional restorations’ mechanical integrity depends on several interacting factors. Optimized filler content enhances strength and hardness while maintaining printability. Print layer thickness inversely affects surface smoothness but has variable effects on strength properties, with optimal thickness being material-dependent. Post-polymerization treatments significantly improve mechanical properties but must be balanced against potential material degradation. Future development should focus on simultaneous rather than independent optimization of these parameters [39,89,92,97].

### 3.3. Physical Properties

#### 3.3.1. Color Stability

Provisional crowns and bridges fulfill essential prosthodontic functions, protecting prepared teeth while maintaining esthetics during interim treatment phases. Their prolonged intraoral presence necessitates sustained color stability for optimal patient satisfaction and treatment outcomes [1,2,128,129]. The susceptibility of provisional restorations to discoloration from common dietary substances significantly impacts clinical success, particularly with the increasing adoption of additive manufacturing in dentistry [1,5,130,131]. Systematic investigations have documented performance variations across fabrication methodologies.

Comparative studies consistently demonstrate inferior color stability of 3D-printed provisional materials relative to conventional and CAD/CAM alternatives. Yildirim et al. reported unacceptable discoloration of printed specimens after coffee immersion [128], while Alalawi et al. observed that NextDent and FormLabs printed resins exhibited significantly greater chromatic alterations than milled (Telio CAD) counterparts following exposure to dietary solvents, particularly coffee and tea [129]. Similarly, evaluations of E-Dent 100 and VeroGlaze revealed clinically unacceptable color differences (ΔE > 3.3) after 8 weeks of beverage exposure, with discoloration patterns correlating with structural vulnerabilities inherent to layer-by-layer fabrication, including compromised interlayer adhesion and elevated water sorption rates [2,128,129].

**Figure 5:**
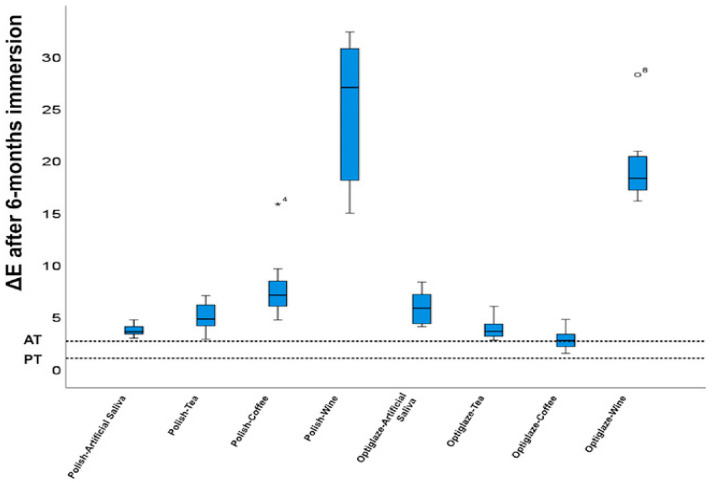
The significance of the color changes on the acceptability threshold (AT) and perceptibility threshold (PT) tested for ΔE after 6-month immersions. AT is defined as 2.7 and PT is defined as 1.0. * Represents ΔE from Haselton et al. (doi: 10.1016/j.prosdent.2004.09.025). The circle Represents ΔE from Tom et al. (doi: 10.15713/ins.jcri.141). Reprinted with permission from [132].

Manufacturing parameters and surface treatments significantly influence the color stability of 3D-printed provisional restorations. Increased layer thickness (100 μm versus 25 μm) demonstrates enhanced chromatic resistance, while printing orientation optimization (particularly 0° versus 45° or 90°) reduces susceptibility to staining agents [1,91]. Surface treatment interventions show promising results, as demonstrated by Almejrad et al., who evaluated NextDent Crown & Bridge specimens with either conventional polishing or nanofilled light-polymerizing protective coating (Optiglaze) after six months of immersion in staining solutions. While red wine induced the most pronounced discoloration in both groups, the Optiglaze-treated specimens exhibited significantly superior color stability against all chromogenic agents, particularly coffee and wine, as shown in Figure 5 [132].

The representative studies on color stability are summarized in Table 3. These findings underscore the critical importance of optimizing manufacturing parameters and implementing appropriate surface treatments to enhance the long-term esthetic performance of 3D-printed provisional restorations in clinical prosthodontic applications.

#### 3.3.2. Water Sorption and Solubility

Water sorption (WS) and solubility (SL) represent critical determinants of dental material longevity and clinical performance [11,63,133]. WS behavior predominantly correlates with polymer matrix hydrophilicity and cross-linking density, where hydrophilic functional groups and residual monomers facilitate water–polymer interactions, resulting in component elution and hydrolytic degradation [134,135]. The integrity of the filler–polymer interface further modulates these properties, as compromised covalent bonding creates micro-voids that serve as infiltration pathways for water molecules, subsequently inducing polymer chain hydrolysis, dimensional instability, and accelerated leaching of unreacted components. These degradative processes collectively compromise mechanical integrity and functional longevity, necessitating compliance with ISO 10477:2020 standards (WS ≤ 40 µg/mm^3^, SL ≤ 7.5 µg/mm^3^) to ensure adequate clinical performance, while recent studies on 3D-printed dental resins have demonstrated water sorption values between 25.31 and 37.94 μg/mm^3^ and solubility values ranging from 0.08 to 8.27 μg/mm^3^ [17,39,135,136,137,138,139]. The representative studies of 3D-printed resin-based dental provisional crown and bridge materials on water sorption and solubility are summarized in Table 4.

Britto et al. evaluated the water sorption (Wsp) and solubility (Wsl) of a 3D-printed resin-based polymer intended for provisional dental crown, comparing it to bis-acryl composite resin (BA) and heat-cured acrylic resin (AR). Results indicated that the 3D-printed polymer exhibited a Wsp of 14.4 μg/mm^3^ and a Wsl of 3.5 μg/mm^3^, both of which were below the maximum limits set by the ISO 10477:2020 standard. In contrast, AR demonstrated the highest Wsp (26.5 μg/mm^3^) and the lowest Wsl (0.6 μg/mm^3^). However, the long-term performance of 3D-printed materials in clinical settings is still limited and further research is needed to validate these findings and explore the full potential of 3D printing in dental applications [39]. Perea-Lowery et al. found that the 3D-printed resins (Imprimo Cure and Form Cure groups) exhibited similar water sorption (2.2%) to conventional materials, but higher water solubility compared to heat-cured alternatives. Heat-cured resins showed the lowest water solubility (0.32%), attributed to their higher processing temperatures and extended curing durations [140].

Water uptake occurs either through penetration into micro-voids or through molecular interactions, depending on the polarity of the resin [140,141]. Various strategies can be implemented to enhance water sorption and solubility performance in denture base materials [84,140,142]. For instance, post-curing optimization plays a vital role through the enhanced cross-linking of unreacted chemical groups, particularly when combining photo and thermal polymerization with extended duration. Processing improvements focus on achieving a higher degree of double-bond conversion and ensuring complete polymerization to minimize residual monomer content [142]. Material composition considerations are equally important, especially through careful attention to resin polarity, available polar sites for hydrogen bonding, and the strategic use of cross-linking agents to improve water resistance [143,144]. These treatments are essential as water absorption and solubility significantly impact the material’s durability and performance in the oral environment [11,63,133,135,136,137].

#### 3.3.3. Surface Roughness

Surface roughness significantly influences the clinical performance of provisional dental restorations, with multiple fabrication parameters affecting this property in 3D-printed materials [94,145]. The primary contributors to surface irregularities in 3D-printed resins include pixel-based curing variations, layer stratification, and print orientation. These factors create characteristic lamination lines and surface imperfections that compromise mechanical integrity and reproducibility. Notably, printing orientation directly modulates surface quality, with perpendicular (90°) orientation demonstrating superior outcomes compared to parallel or angular alternatives (0°, 15°, and 45°) [86,94,145]. Layer thickness constitutes another critical determinant, with thinner layers (25 μm) producing significantly smoother surfaces (Ra = 0.17–0.2 μm) that remain below the clinically relevant threshold for bacterial colonization (Ra ≤ 0.2 μm) while simultaneously optimizing translucency and color matching. Conversely, increasing layer thickness to 50 μm or 100 μm progressively deteriorates surface quality (Ra = 0.23–0.79 μm), reduces translucency, and introduces polymerization heterogeneity with increased void formation [63,146,147]. The representative studies of 3D-printed resin-based dental provisional crown and bridge materials on surface roughness are summarized in Table 5.

**Figure 6:**
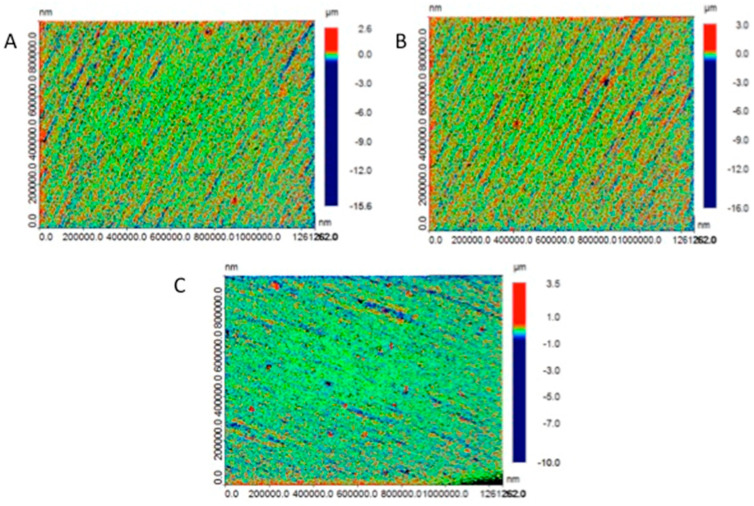
Surface roughness micrographs for study samples in (**A**) conventional, (**B**) CAD/CAM, and (**C**) 3D printing groups [86].

Material composition significantly influences surface characteristics, with methacrylate-based formulations generally exhibiting superior surface smoothness compared to bis-acryl alternatives due to enhanced filler–matrix integration and more predictable polymerization kinetics [148]. Post-processing protocols substantially impact final surface quality, with extended curing times and professional polishing sequences demonstrating up to 50% reduction in Ra values. These interventions are particularly important for 3D-printed restorations, which generally exhibit greater inherent surface roughness (Ra = 5.61 ± 0.33 μm) compared to CAD/CAM milled alternatives (Ra = 3.28 ± 0.34 μm), with hand-mixed provisional materials demonstrating intermediate values (Ra = 4.43 ± 0.41 μm), as shown in Figure 6 [86,149]. The significant variations in surface quality across manufacturing methodologies necessitate careful consideration of printing parameters and post-processing protocols to optimize clinical outcomes.

**Table 5:** Summary of surface roughness from representative reviewed studies.

Author and Year	Evaluated Materials	Specimen Fabrication Technique	3D Printing Parameters	Key Results
Aldahian et al. (2021) [86]	-Conventional: PMMA -CAD/CAM: PMMA blocks -3D Printing: Dimethacrylates	-Conventional: Auto-polymerizing resin poured into molds -CAD/CAM: Milled from PMMA blocks using a wet milling machine -3D Printing: SLA	-Layer thickness: 50 μm -Wavelength: 405 nm -Curing time per layer: 2.40 s -3D-printed specimens soaked in 99% isopropyl alcohol for 60 s	-Highest surface roughness observed in 3D-printed specimens (5.61 ± 0.33 μm). -Lowest surface roughness observed in CAD/CAM specimens (3.28 ± 0.34 μm).
Wadhwani et al. (2022) [150]	NextDent C&B temporary tooth-colored resin (SprintRay for DLP and FormLabs for SLA)	3D printing using SLA and DLP technologies	-Specific values of layer thickness not provided -Cleaning with 99.9% ethyl alcohol, clipping support structures, and light curing according to manufacturer’s guidelines	Both SLA and DLP samples showed surface roughness within acceptable ranges. Polishing significantly reduced roughness depth (*p* < 0.05). SLA samples had slightly lower roughness than DLP.
Rafat Sasany et al. (2024) [146]	-VarseoSmile Crown Plus-Crowntec: Similar to VS but with pyrogenic silica -NextDent C&B MFH -GC Temp PRINT	DLP	-Layer thickness (μm): 25, 50, 100 -Build angle: 30 degrees -Post-print cleaning with alcohol -Specimens were polished with silicon carbide papers (500–4000 grit) and stored in the dark	-Surface roughness (*Ra*) increased significantly with 100 μm layer thickness (*p* ≤ 0.001). -At 25 μm layer thickness, *Ra* values were within the 0.2 μm threshold for all materials.
Shin et al. (2020) [147]	-Polycarbonate block: Polycarbonate, nanosilica filler, glass fiber, alkoxysilane -PMMA: High cross-linked PMMA resin -Dispersed–Filled Composite: Composite resin material (BisGMA, TEGDMA) with 77 wt% silica, zirconia, and barium glass nanoparticles -NextDent C&B -Denture Teeth A2 resin	-CAD/CAM blocks: Milled using a precision cutting machine -3D printing resins: DLP and SLA	-Layer thickness: 100 µm -Printers: DLP (NextDent ND5100) and SLA (Form 3) -Settings: 405 nm UV LED light and 250 mW laser power -Washed using 90% isopropyl alcohol -Post-curing conducted in UV post-curing equipment	-3D printing resins showed smoother surfaces compared to CAD/CAM blocks, which had rough surfaces with traces of bur passes.

Surface roughness optimization remains essential for maximizing the esthetic integration, minimizing bacterial adhesion, enhancing marginal adaptation, and extending the functional longevity of provisional restorations [85,143]. Achievement of clinically acceptable outcomes requires systematic consideration of multiple interdependent variables, including orientation, layer parameters, material selection, and post-processing protocols. These factors must be evaluated collectively rather than in isolation to develop standardized, evidence-based manufacturing protocols that consistently produce provisional restorations with optimized surface characteristics. This integrated approach is particularly critical for advancing the clinical application of additive manufacturing technologies in contemporary restorative dentistry, where surface quality directly impacts both biological compatibility and prosthetic performance.

To summarize, the physical properties of 3D-printed provisional materials exhibit both advantages and limitations compared to conventional alternatives. Water sorption (typically 2–3%) remains comparable to traditional materials, though some formulations demonstrate higher solubility. Surface roughness consistently presents a challenge, with Ra values ranging from 0.2 to 5.6 μm depending on processing parameters, which can be mitigated through optimized post-processing. Critical factors affecting physical stability include post-curing optimization, layer thickness (which significantly impacts surface characteristics and should be minimized when esthetics are prioritized), and printing orientation (with 90° orientation generally yielding superior results).

#### 3.3.4. Marginal Adaptation and Internal Fit

In recent years, 3D printing has been adopted in dentistry to produce patient-specific dental implants quickly, though with slightly lower but still acceptable accuracy compared to conventional methods [33,151]. High-quality provisional crowns are essential for protecting prepared teeth and maintaining periodontal health [130,152]. They also preserve the functional and esthetic integrity of the oral cavity. The marginal adaptation and internal fit of restorations are critical factors for ensuring their clinical success. Poor marginal fit can lead to microleakage and plaque accumulation, increasing the risk of cement degradation, secondary caries, and periodontal complications [153,154]. Achieving precise marginal adaptation is essential for enhancing restoration durability and performance of provisional crown and bridge. The representative studies of 3D-printed resin-based dental provisional crown and bridge materials based on marginal adaptation and internal fit are summarized in Table 6.

The accuracy of marginal fit is a critical factor in the success of dental restorations and is influenced by multiple variables, including the quality of tooth preparation, the precision of impression techniques, the selection of restorative materials, the fabrication technology utilized, and the type of luting cement applied. A good marginal fit helps maintain periodontal health. It reduces cement dissolution, lowers the risk of microleakage, and prevents problems like secondary caries and periodontal inflammation [150,155]. For instance, Wadhwani et al.’s comparison of the marginal adaptation of SLA- and DLP-printed resins indicated that SLA samples exhibited significantly better marginal adaptation compared to DLP samples. Specifically, the mean marginal gap for SLA samples was 49.6 μm for the first molar and 106.8 μm for the first premolar, while DLP samples had mean marginal gaps of 101.8 μm for the first molar and 157.5 μm for the first premolar. The study found that all DLP samples showed a statistically significant higher mean marginal gap compared to SLA samples (*p* < 0.005), suggesting that SLA-printed provisional restorations provide a superior fit, which is critical for the longevity and effectiveness of dental restorations [150].

Cakmak et al. evaluated the impact of different printing layer thicknesses (20 μm, 50 μm, and 100 μm) on the trueness and marginal quality of 3D-printed interim dental crowns in comparison to conventionally milled PMMA crowns. The findings revealed that crowns printed with 20 μm and 50 μm layer thicknesses exhibited trueness comparable to milled crowns, whereas a 100 μm layer thickness resulted in significantly reduced trueness and inferior marginal quality. Among the tested parameters, a 50 μm layer thickness provided the optimal balance between quality and efficiency. However, the study was limited to a single material, restricting the generalizability of the results to other materials. The research demonstrated that 3D printing is a viable method for fabricating provisional crowns when appropriate layer thicknesses (20–50 μm) are used, although marginal quality varied depending on the crown’s location. This study offers valuable insights into the clinical application of 3D printing for provisional dental restorations and underscores the importance of optimizing printing parameters to achieve satisfactory outcomes [156].

Generally, marginal discrepancies were least with the provisional restorations fabricated by 3D printing when compared to those fabricated by CAD/CAM milling and conventional techniques [157,158,159]. For example, Ahlholm et al. found that 3D-printed mold restorations exhibited significantly lower gap values (44.3 μm for marginal gaps) than milled restorations (58.4 μm), with internal gaps being 6% to 51% smaller. These results were within clinically acceptable limits, typically defined as less than 120 μm, though some studies allow for a maximum misfit of up to 200 μm [157]. The discrepancies in accuracy may result from variations in thickness and layer shrinkage, particularly along the Z-axis [160]. Additionally, the type of finish line used in crown preparation also influenced marginal discrepancies. Studies found that a rounded shoulder with a bevel finish line produced the least marginal discrepancy, while chamfer and knife-edge finish lines showed higher discrepancies. However, the fabrication method had a greater impact on marginal fit than the type of finish line [155,158,159].

**Table 6:** Summary of marginal adaptation and internal fit from representative reviewed studies.

Author and Year	Evaluated Materials	Shape and Dimension of Tested Resin Samples	Key Results
Wadhwani et al., 2022 [150]	-NextDent C&B temporary tooth-colored resin	3-unit fixed partial denture (FPD)	-SLA technology demonstrated better marginal adaptation compared to DLP; the study suggests SLA might be more precise for dental provisional restorations
Pekka Ahlholm et al., 2024 [157]	-Resin: G-aenial Universal Injectable (GC) -Flowable Composite: everX Flow (GC)	12 different restorations: -11 inlays/onlays -1 crown Approximate dimensions: 3.5–7.7 mm width, 6–10 mm height, 1.6–4.2 mm depth	-Internal gaps in the 3D-printed mold group were 6% to 51% smaller than in the milled group -The accuracy was more favorable for most restoration types, except for crown preparations -3D-printed mold restorations showed better fit in complex shapes and long margins
Saurabh Chaturvedi et al. (2020) [159]	-Protemp™ 4 (3M ESPE, Seefeld, Germany) -Formlabs Dental SG Resin (Formlabs Inc., USA) -Ceramill TEMP (Amann Girrbach, Maeder, Austria)	Provisional crowns for maxillary first premolar with three finish-line designs: -Chamfer -Rounded shoulder -Rounded shoulder with bevel	-3D-printed crowns showed minimal marginal gap -Best overall internal fit compared to molding and milling methods
Nawal Alharbi et al. (2018) [158]	Temporis™ (Hybrid composite resin material)	Maxillary central incisor crown models	-3D-printed restorations showed significantly lower marginal and internal gaps compared to milled restorations
Tahayeri et al. (2018) [15]	1. NextDent C&B (3D-printed resin)—Vertex Dental 2. Integrity^®^—Dentsply Canada Ltd., Woodbridge, Canada 3. Jet^®^—Lang Dental Inc., Wheeling, IL, USA	Test bars: 25 mm × 2 mm × 2 mm	Printing accuracy varied as follows: -Length: 0.12–2.4% error -Width: Up to 22% error -Thickness: Up to 20% error -90° orientation and white resin setting were most accurate
Peng et al., 2020 [161]	1. Jet (Lang Dental Inc., Wheeling, IL, USA)—Autopolymerized PMMA resin, APP group 2. ZCAD Temp Fix (Harvest Dental)—CAD/CAM PMMA resin, CAM group 3. NextDent C&B MFH (3D Systems)—3D-printed methacrylic oligomers, 3DP group	Interim dental crowns on a mandibular first molar resin die	1. Digitally fabricated crowns (CAM and 3DP) had significantly better internal fit and smaller marginal discrepancy compared to manually constructed crowns 2. No significant difference between CAD/CAM and 3D-printed crowns 3. Gap distance ranged from 0.13 mm to 0.55 mm 4. Cement space volume ranged from 24.09 to 33.67 mm^3^
Wan-Sun Lee et al., 2017 [162]	1. Vipi block (VIPI, Pirassununga, Brazil)—Milling block 2. VeroGlaze MED620 (Stratasys, Minnetonka, MN, USA)—3D Printing Resin 3. ZMD-1000B (Dentis, Daegu, Korea)—3D Printing Resin	Interim dental crowns for upper first molar	-Mean discrepancy: CAD/CAM milling: 171.6 (97.4) μm 3D printing (Stratasys): 149.1 (65.9) μm 3D printing (Dentis): 91.1 (36.4) μm -3D printing showed statistically better fit than milling

Nevertheless, a good internal fit ensures a proper and even luting space while maintaining retention and resistance during cementation [145,163,164]. The internal adaptation of 3D-printed provisional crowns and fixed dental prostheses (FDPs) is generally superior to that of restorations fabricated using CAD/CAM milling due to several key factors [15,40,155]. For example, Peng et al. found that 3D-printed crowns demonstrated internal fit and marginal discrepancies comparable to those of CAD/CAM crowns, both of which outperformed manually constructed crowns [161]. The incremental layering process of 3D printing allows for the precise reproduction of intricate dental anatomy and effectively compensates for polymerization shrinkage, unlike the subtractive nature of CAD/CAM milling, which can introduce inaccuracies. Additionally, 3D printing materials, such as methacrylate oligomers and hybrid composites, exhibit lower volumetric shrinkage and superior mechanical properties compared to conventional PMMA used in milling [165,166,167,168]. The automated and additive nature of 3D printing also minimizes distortion and human error, ensuring greater consistency and accuracy in the fit of restorations. Advanced measurement techniques, such as micro-CT scanning, further improve the evaluation of 3D-printed restorations, often providing more detailed and reliable assessments than traditional methods used for CAD/CAM milled restorations [155,165,166,167,168]. For instance, Lee et al. evaluated the internal fit of interim crowns manufactured using CAD/CAM milling and two types of 3D printing methods, focusing on the accuracy of provisional restorative resins. The 3D-printed crowns exhibited significantly lower mean discrepancies (149.1 μm and 91.1 μm for the two printing methods) compared to the milling method (171.6 μm), highlighting the superior marginal and internal fit of 3D printing as a promising alternative for dental prosthesis production [162].

In brief, the combination of advanced fabrication techniques, favorable material properties, and reduced human error in 3D printing contributes to its enhanced marginal adaptation and internal adaptation of 3D-printed provisional crowns, which typically exhibit superior marginal fit and internal adaptation, establishing them as a reliable alternative in dental prosthetics [150,153,154,161,162,169].

#### 3.3.5. The Effect of pH on the Durability of 3D-Printed Resins

The oral environment exhibits considerable pH fluctuations due to dietary habits, bacterial metabolism, and salivary composition, significantly affecting the durability of dental materials, particularly resin-based provisional restorations. Acidic conditions induce clinically detectable surface roughness and dullness in restorative materials, while resin-based materials undergo softening when exposed to plaque acids, food-simulating agents, and enzymes. This degradation mirrors the well-documented erosive effects on dental hard tissues [131,170,171,172]. Specific beverages exert compound effects through multiple mechanisms; coffee’s solvent agents and tannic acid reduce pH, increase surface solubility, and cause discoloration [172,173], while red wine affects 3D-printed resins through its acidity, colorants, and alcohol content [91].

Research findings provide quantifiable evidence of pH-dependent degradation in additively manufactured dental materials. Alalawi et al. demonstrated that acidic conditions (pH 5.7) reduced microhardness in both 3D-printed resins and milled PMMA, though less severely than complex solutions such as coffee or tea. Neutral or basic pH environments minimally affected hardness and color stability, suggesting staining agents contribute more significantly to degradation than pH-dependent hydrolysis alone [129]. Lee et al.’s investigation revealed distinctive discoloration patterns in 3D-printed resins exposed to acidic solutions (coffee pH 4.6, wine pH 3.5), with coffee showing peak discoloration at 15 days followed by a decline, while wine caused continuous discoloration progression. Notably, manufacturing parameters significantly influenced degradation susceptibility, with thicker layers (100 μm) and 0° orientation demonstrating 34% less coffee-induced discoloration than thinner counterparts [91].

The underlying mechanism for these observations involves hydrolytic degradation of methacrylate-based resins under acidic conditions, resulting in surface softening, increased water sorption, and compromised mechanical integrity. While low pH environments significantly increase surface hardness loss in acrylic materials, they exert minimal effects on surface roughness parameters [91,172,173]. These findings have direct clinical implications, indicating that when selecting provisional restorative materials, resistance to acidic degradation should be prioritized to ensure optimal performance in the variable oral environment. Additionally, manufacturing parameters such as layer thickness and orientation may be optimized to enhance resistance to discoloration and degradation in acidic conditions, particularly for long-term provisional restorations.

### 3.4. Biocompatibility

Biocompatibility is a critical consideration for 3D-printed provisional dental resins, as these materials are used in direct contact with oral tissues and must ensure patient safety while supporting tissue integration [174,175]. Comprehensive biocompatibility assessment according to the ISO 10993 series standards encompasses multiple critical parameters: cytotoxicity, genotoxicity, allergenicity, inflammatory response, and reproductive toxicity [175,176,177,178,179,180]. Current standard evaluation protocols include in vitro cell viability assays (MTT, SRB, Live/Dead staining), inflammatory marker quantification (IL-6, PGE2), oxidative stress measurement (GSH/GSSG ratio), and apoptosis detection [175,181,182]. Recent research has extensively evaluated the biocompatibility of 3D-printed dental resins, consistently demonstrating their safety and effectiveness as materials for provisional restorations, which are as summarized in Table 7 [39,61,88,181].

Atria et al. assessed the biocompatibility of three commercial 3D-printed resins and one experimental resin (Permanent Bridge), finding no significant differences in cell proliferation across materials and time points. Notably, NextDent exhibited higher initial cell proliferation (86.1%) at 24 h compared to other materials (63–65%), with minimal cytotoxicity (13.7%) that remained within acceptable limits [181]. Britto et al. evaluated the biocompatibility of 3D-printed polymers using SRB and MTT assays, reporting superior cell viability (92.88% ± 11.36%) for 3D-printed resins compared to heat-cured acrylic resin (71.94% ± 11.82%) and comparable performance to bis-acryl resin (90.85% ± 11.60%). All tested materials maintained cell viability above the acceptable threshold after 72 h [39]. Furthermore, another study compared six resin materials, including four 3D-printed resins, one subtractive material, and one conventional material, revealing that most 3D-printed resins exhibited better biocompatibility than conventional materials, with Luxatemp and 3Delta temp significantly reducing cell viability [61]. Collectively, these findings highlight the excellent biocompatibility of 3D-printed resins, provided manufacturer protocols are followed, making them viable alternatives for temporary dental restorations [39,61,181].

The addition of fillers, such as glass silica and zirconia nanoparticles, to 3D-printed dental resins also demonstrated significant potential in improving biocompatibility. The incorporation of fillers reduces the proportion of the organic phase in the resin, thereby decreasing the amount of unpolymerized monomers that could negatively impact cell viability [45,107,108,109,183]. For instance, Alshamrani et al. evaluated the biocompatibility of 3D-printed dental resins reinforced with varying concentrations (5%, 10%, and 20%) of glass silica and zirconia nanoparticles using MTT assays and Live/Dead cell staining. Results showed that all tested groups exhibited cell viability above 80%, with the zirconia 10% group achieving the highest cell viability, indicating non-cytotoxicity. Zirconia-reinforced resins consistently outperformed glass silica groups in cell viability, likely due to more uniform filler distribution and reduced unpolymerized monomer content. However, the use of different-sized nanoparticles posed a limitation, complicating the evaluation of individual effects on resin properties. As a whole, incorporating zirconia and glass fillers creates biocompatible 3D-printed resins suitable for dental applications, particularly for temporary crown restorations, though further research is needed to explore long-term clinical implications [45].

Wuersching et al. systematically investigated the initial biocompatibility of novel printable resins through multiple interconnected factors, providing a more comprehensive view of the biological impact of dental resins [175]. Most printable resins exhibited moderate to severe cytotoxicity, with VarseoSmile Temp showing particularly severe toxicity resulting in nearly 0% cell survival after 72 h. In terms of inflammatory response, while most materials maintained IL-6 levels similar to the control group, VarseoSmile Crown plus and P Pro Crown & Bridge notably increased PGE2 levels. The study identified elevated oxidized glutathione levels in several temporary FDP materials, particularly VarseoSmile Temp and P Pro Crown & Bridge. Several factors contributed to these results, including the lower filler content (<50% by weight) in printable resins, the presence of photoinitiators (TPOs) in all five printable materials, and the manufacturing method and polymerization process. However, the research indicated that biocompatibility could be enhanced through post-processing steps such as UV curing at 60 °C, and suggested additional measures like extra curing, washing, and immersing 3D-printed restorations in liquid before oral insertion to allow toxic components to elute. While printable resins demonstrated higher cytotoxicity compared to traditional materials, the study also concluded that their biocompatibility could be significantly improved through optimized post-processing procedures [175].

**Figure 7:**
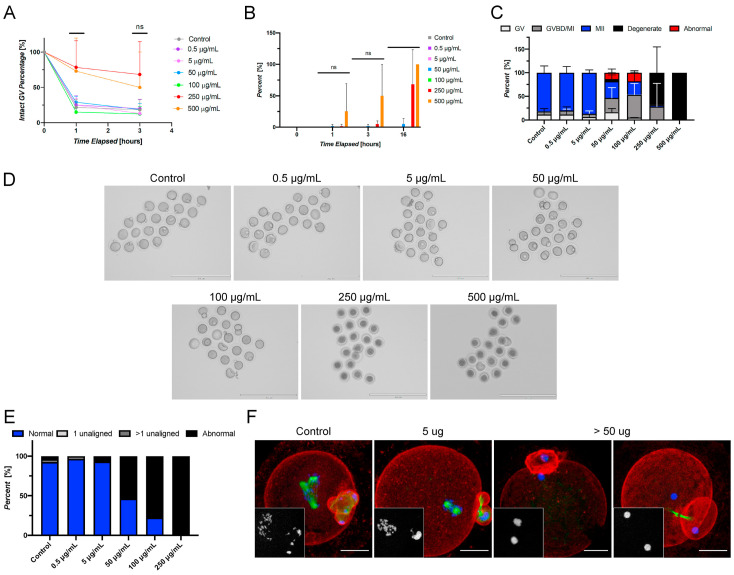
Tinuvin 292 induces abnormal meiotic phenotypes and oocyte degeneration in a dose-responsive manner in vitro. (**A**) The percentage of oocytes with intact germinal vesicles at 0, 1, and 3 h of culture in each material showing altered meiotic kinetics, as evidenced by an increased percentage of the GV-intact phenotype in oocytes exposed to concentrations of 250 and 500 μg/mL. (**B**) The percentage of oocytes determined to be degenerate at 0, 1, 3, and 16 h, showing rapid degeneration of oocytes matured in the presence of 250 and 500 μg/mL. (**C**) The resulting meiotic progression rates of oocytes matured in the presence of Tinuvin 292, showing meiotic disruption at concentrations of ≥50 μg/mL. (**D**) Representative images of oocytes following IVM in the presence of a range of concentrations of Tinuvin 292. Scale bar: 200 μm. (**E**) The average incidence rates of chromosomal abnormalities showing the dose–response effect of Tinuvin 292. (**F**) Representative confocal microscopy images of abnormal phenotypes observed in control, 5 μg/mL, and the 50, 100, and 250 μg/mL conditions. The insets show DNA. Scale bar: 25 μm. The error bars represent the standard deviation. Statistical significance was determined using a one-way ANOVA (ns = not significant). Three technical replicates of IVM were performed with between 19 and 20 oocytes/dose, for a total of 459 oocytes used in this experiment [176].

Similarly, Rogers et al. investigated the reproductive toxicity of two ISO-certified “biocompatible” 3D printing resins (Dental SG/DSG and Dental LT/DLT) on mammalian oocytes. The research reveals that exposure to DLT resin leads to significantly higher oocyte toxicity than DSG. The results showed that the light stabilizer Tinuvin 292 leaches from DLT resin, contributing to meiotic disruption and increased chromosomal abnormalities in oocytes, as shown in Figure 7. Although oxygen plasma treatment improved outcomes for DSG, 57% of oocytes nonetheless displayed abnormal morphology. The findings emphasize the critical need for thorough assessments of materials labeled as biocompatible, particularly regarding their safety in reproductive health applications, advocating for stricter evaluation standards when developing biomedical devices. The work advocates revising biocompatibility frameworks to address leachable compounds’ systemic impacts [176].

These results consistently demonstrate that 3D-printed resin is a safe and viable alternative for provisional crown and artificial teeth applications. However, while the findings confirm the adequate biocompatibility of these materials, further comprehensive studies are required to fully characterize the biological properties of emerging 3D printing materials, especially for broader dental applications. Such research is crucial for developing standardized protocols and ensuring their long-term clinical safety and effectiveness [39,45,61,88,176,181].

### 3.5. Antibacterial Property

Dental restoration materials, such as provisional crown and bridge, often create saliva retention zones due to imperfect adaptation to tooth surfaces. These interfacial microgaps facilitate bacterial proliferation and biofilm formation, which may damage both the restoration materials and the tooth and then lead to restoration failure. To address this challenge, contemporary research focuses on enhancing conventional dental materials with antimicrobial functionality [48,184,185]. Current strategies incorporate antibacterial additives such as fluorides, zinc ions, antimicrobial peptides, quaternary ammonium salts, and chlorhexidine into resin matrices [184,186,187,188]. The development of antibacterial 3D-printed resin-based provisional crowns and bridges also demonstrates particularly promising results, with key findings summarized in Table 8 [184,189,190,191,192].

For instance, the addition of Ag-loaded halloysite nanotubes (Ag-HNT) to stereolithography resins has demonstrated dual benefits: sustained antibacterial efficacy against *Streptococcus mutans* (reducing biofilm formation by 60–75%) and a 25% improvement in bending strength [184]. Similarly, titanium dioxide (TiO_2_) nanoparticles have shown broad-spectrum antimicrobial activity, achieving 90% inhibition of *Candida albicans* [189].

Advanced approaches employ quaternary ammonium salt monomers as copolymerized antimicrobial constituents [191,192,193]. For instance, Li et al. developed a DLP 3D printing system incorporating two quaternary ammonium compounds—QAC (methacrylate-based small molecule) and SH-QAC (thiol-functionalized oligomer)—into a ternary matrix resin. These agents covalently bond to the polymer network during photopolymerization, providing contact-based antimicrobial activity without leaching. The system showed excellent efficacy, with 4 wt% QAC effective against both *E. coli* and *S. aureus*, while 10 wt% SH-QAC targeted *S. aureus*. In addition, the formulation achieved rapid curing (>85% conversion in 10 s) with reduced volume shrinkage (8.67%). However, QAC addition compromised performance, reducing thermal stability (60 °C decrease in decomposition temperature at 8 wt%) and tensile strength (25.8 to 6.6 MPa). While QAC showed sedimentation issues, SH-QAC improved compatibility, suggesting potential applications in dental prosthetics like provisional crowns and bridges [192].

**Figure 8:**
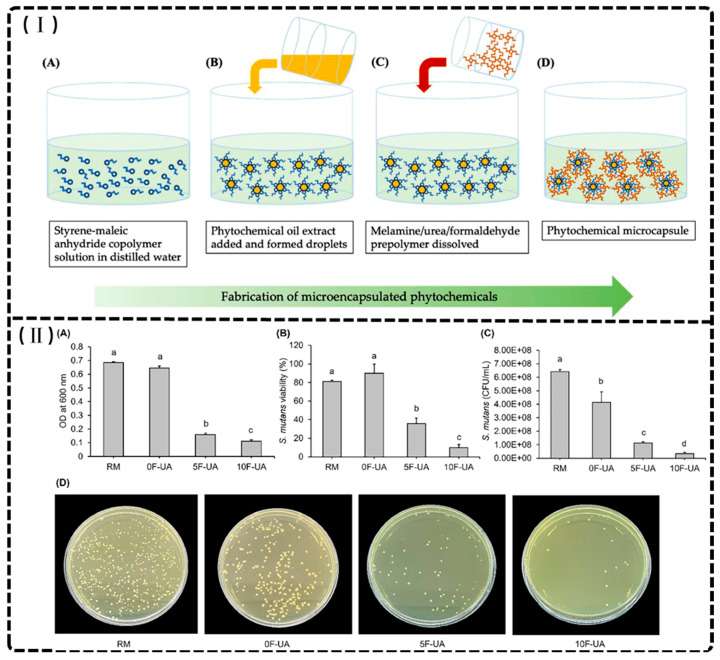
(**I**) Schematic flowchart of microencapsulation of phytochemicals. Reprinted with permission from [190]. (**II**) Results of direct contact tests. (**A**) OD values at 600 nm, (**B**) *S. mutans* viability in WST-8 assays, (**C**) CFU counts. Different lower-case letters indicate significant differences (*p* < 0.05). (**D**) Images of *S. mutans* colonies in direct contact tests for RM, 0F-UA, 5F-UA, and 10F-UA groups. Reprinted with permission from [194].

Phytochemicals serve multiple roles in oral health, extending beyond analgesic and anti-inflammatory effects to include microbial biofilm control [188,190]. Jo et al. investigated the efficacy of microencapsulated phytochemicals (MPs, as shown in Figure 8I), specifically (-)-α-pinene from phytoncide oil, when incorporated at 5% (*w*/*w*) into 3D-printed dental polymers. The MPs significantly reduced microbial growth and biofilm formation (*p* ≤ 0.001) while maintaining biocompatibility with human gingival fibroblasts (*p* = 0.310). Treatment increased surface roughness (*p* < 0.001) and hydrophobicity (contact angle 81.83° vs. 76.31° in controls). These results position MPs as a promising antimicrobial solution for 3D-printed dental prostheses, but limitations require consideration including unvalidated clinical efficacy and potential durability impacts from increased surface roughness [190].

Novel approaches like fluoride-releasing urethane acrylate (UA) resins further enhance functionality by combining mechanical stability (flexural strength > 80 MPa) with long-term fluoride release (28-day cumulative release ≈ 1.2–1.8 μg/cm^2^), effectively inhibiting cariogenic bacteria while maintaining cytocompatibility with oral tissues. Results of direct contact tests are as shown in Figure 8II. These materials leverage 3D printing’s precision to optimize nanoparticle distribution, ensuring uniform antimicrobial action without compromising structural integrity. Despite the study’s limitations, dental prostheses such as crowns, bridges, or orthodontic appliances fabricated from this resin may enable sustained fluoride release to adjacent teeth [194].

However, challenges persist in balancing antibacterial efficacy with material durability and long-term biocompatibility of these resins. While in vitro studies report 70–90% bacterial reduction, clinical validation is limited by insufficient data on leaching kinetics and potential cytotoxicity of residual monomers [184,189,190,191,192,194]. On the other hand, the highly cross-linked polymeric structure of 3D-printed dental resins results in materials that are barely biodegradable and hard to recycle, so most prints are disposed of by landfilling or incineration—practices that entail significant environmental burden. The use of certain light stabilizers and additives, such as Tinuvin 292, introduces further ecological and health concerns: the leaching of these compounds can result in documented reproductive and cytotoxic effects. These findings highlight the importance of robust post-processing protocols and careful resin formulation to minimize potential risks [176]. Future research should prioritize multi-functional resin formulations that synergize antimicrobial, mechanical, and esthetic properties while addressing polymerization-induced stress and interlayer adhesion weaknesses. Standardized protocols for evaluating clinical performance under dynamic oral conditions are essential to translate these innovations into routine practice.

## 4. Clinical Applications

### 4.1. Clinical Workflow

The clinical workflow for the fabrication of 3D-printed provisional crowns generally involves several key steps, utilizing digital technology to enhance efficiency and accuracy [21,28,195,196,197].

**Figure 9:**
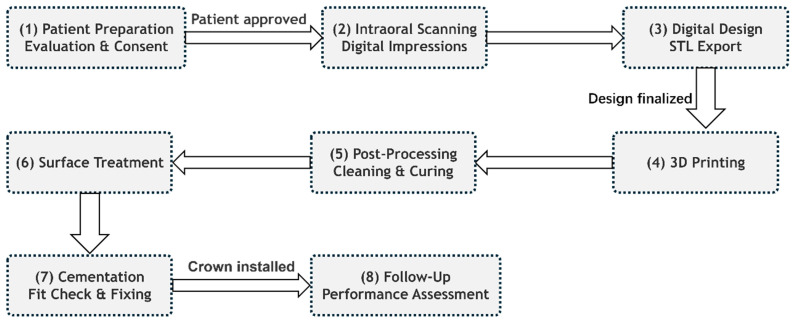
Clinical workflow of 3D printing dentistry for provisional crowns and bridges.

As shown in Figure 9, the clinical workflow for 3D-printed provisional crowns and bridge begins with patient eligibility assessment and informed consent, followed by digital impression acquisition using an intraoral scanner to capture the dental arch and implant position. The scanned data are then exported as STL files for digital design using specialized CAD software, where the crown is designed to match adjacent teeth with appropriate spacing parameters. The design is then manufactured using a 3D printer with specific printing parameters (e.g., build angle, resolution) using 3D print resin. Post-processing involves multiple steps: support removal, cleaning, air drying, and UV curing, followed by surface enhancement through glaze application and final UV curing. The completed provisional crown undergoes clinical fitting to verify proper adaptation, including evaluation of fit accuracy, interproximal contacts, and occlusal adjustment, before being cemented with temporary cement. The entire process typically requires two appointments due to the time needed for digital planning, printing, and post-processing procedures, with follow-up assessment to monitor restoration integrity and patient satisfaction [21,195,196]. Furthermore, the introduction of digital workflows in dental 3D printing significantly reduces environmental impacts. By eliminating traditional impression materials and enabling in-office, on-demand production, the technology reduces both transportation-associated emissions and the need for auxiliary clinical supplies. Such workflows contribute to improved patient comfort, greater production efficiency, and lower overall resource use in clinical settings. Despite demonstrated biocompatibility and mechanical reliability of printed provisionals, careful attention to resin formulas is warranted; the choice of photoinitiators and diluents not only affects printability and mechanical strength but also has implications for potential toxicity and long-term health impacts [19,198].

### 4.2. Clinical Studies and Evidence

Previous in vitro studies have examined key properties of 3D printing materials, including mechanical strength, wear resistance, color stability, cytotoxicity, and biofilm formation [7,17,199]. While these tests have yielded promising results and provided valuable insights into material properties, they cannot adequately simulate real-world clinical conditions [21,195,200]. Consequently, clinical evidence supporting 3D-printed restorations remains limited [10,21,101,196,201].

In addition, clinical recommendations for commercial 3D-printed restoration materials often rely on manufacturers’ commercial data rather than scientific evidence from laboratory and clinical studies, limiting our understanding of their performance in clinical settings. Moreover, while digital workflows are purported to enhance patient comfort and convenience compared to conventional procedures, this claim lacks robust supporting evidence [21,202,203]. The absence of controlled clinical studies comparing provisional single crowns produced through conventional and 3D-printed workflows represents a critical gap requiring urgent attention [21,204].

**Figure 10:**
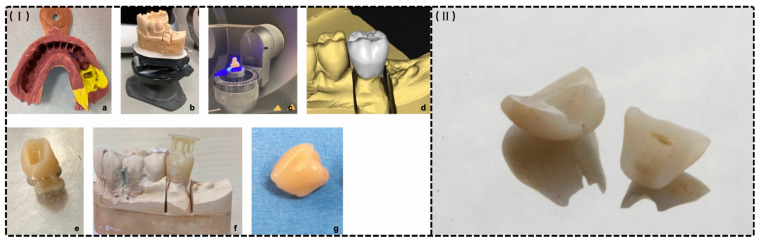
(**I**) An exemplary production process of a 3D-printed temporary crown. (**a**). Conventional impression (**b**). Plaster model (**c**). Digitalization with a laboratory scanner (**d**). Designed restoration in a virtual model (**e**). Three-dimensional printed restoration with resin residues and support structures (**f**). The test fitting of non-finished restoration on the plaster model (**g**). A finished 3D-printed temporary crown [201]. (**II**) The longitudinal fracture of a 3D-printed provisional single crown. Reprinted with permission from [21].

Del Hougne et al. evaluated 3D-printed temporary crowns fabricated with Permanent Crown Resin using a Form3B printer in 63 patients (98 crowns) over 256 days on average (the production process is as shown in Figure 10I). The study was approved by the Institutional Review Board of the University of Würzburg, Germany, and granted permission to use anonymized patient data from the Department of Prosthodontics for the observation period (1 December 2021 to 30 June 2023; approval code 20230710 01). Clinically, the crowns demonstrated a 98% survival rate with only two catastrophic failures, significant improvement in oral health-related quality of life (OHIP scores improved from 6.63 to 2.21, *p* = 0.005), and high patient satisfaction with esthetics (*p* < 0.001). While anatomical form, secondary caries, and sensitivity results were favorable, some issues were noted with surface texture (21% “bravo” ratings) and marginal inflammation (79% “bravo”). These findings support the clinical applicability of 3D-printed temporary crowns, particularly for cases involving teeth with uncertain prognosis or vertical dimension assessment, while recognizing the need for further long-term and risk-specific data [201].

On another hand, De Souza et al. conducted a randomized clinical trial comparing conventional PMMA and 3D-printed provisional single crowns for anterior implants. The study involved 42 crowns (21 per group) placed in 33 patients. This study adhered to the Declaration of Helsinki principles for ethical human research, received approval from Hacettepe University’s Local Ethics Committee (GO 22/57), and was registered with ClinicalTrials.gov (NCT05194293) to ensure transparency. All participants received comprehensive information about study procedures and provided written informed consent before enrollment. Clinical outcomes were similar between groups except for fracture rates: 3D-printed provisionals sample had a significantly higher rate of catastrophic failure (19% vs. 0%, *p* = 0.05). The longitudinal fracture of a 3D-printed provisional single crown was as shown in Figure 10II. However, 3D-printed crowns reduced chairside procedure time to 5:24 min compared to 19:42 min for conventional crowns (*p* < 0.001). Patient satisfaction with esthetics, speech, chewing, and comfort was comparable in both groups. The study demonstrated the efficiency of 3D printing in relation to fabrication speed but emphasized the need for improved fracture resistance and noted that time-consuming post-processing still limits true chairside delivery. Additionally, reliance on single-item VAS measures may not capture the full scope of patient satisfaction [21].

Giannetti et al. compared the occlusal precision of 3D-printed versus milled provisional crowns made from polymethylmethacrylate (PMMA) using the same digital CAD design. This study, approved by the University Federico II of Naples Ethics Committee (Protocol AOU 0024574/19) and registered with ClinicalTrials.gov (NCT04439227), followed Helsinki Declaration principles and STROBE guidelines. A total of 34 patients received both types of crowns, which were scanned before and after occlusal adjustments to assess their trueness. The 3D-printed crowns exhibited significantly lower occlusal differences compared to the milled crowns, requiring less adjustment after placement, demonstrated better dimensional accuracy and potentially reducing chairside adjustments of 3D-printed crowns, thus improving clinical outcomes. However, the study evaluated only three reference points and the use of a single type of 3D printer and milling machine, which may not be generalizable to other systems. The findings support the application of 3D printing technology in the fabrication of temporary crowns, suggesting a promising direction for future research in digital dentistry [204].

These studies collectively underscore the emergence of digital dentistry and the integration of additive manufacturing technologies into clinical practice, suggesting that 3D-printed provisional restorations represent a viable alternative to conventional fabrication methods, contingent upon improvements in material durability for long-term clinical applications [21,204]. On another hand, digital workflows for additively manufactured provisionals offer significant environmental advantages by eliminating physical impression materials and facilitating chairside, on-demand production, thereby reducing transportation-associated emissions and decreasing auxiliary supply consumption. However, concerns regarding incomplete polymerization, which may result in residual photoinitiators or stabilizers with potential for oral leaching, present important considerations for both patient safety and ecological impact that warrant further investigation [61,194].

### 4.3. Limitations and Clinical Challenges

Despite promising outcomes, 3D-printed provisional restorations present several significant limitations affecting clinical implementation. Mechanical reliability remains concerning, with De Souza et al. reporting substantially higher catastrophic failure rates for 3D-printed anterior implant provisionals compared to conventional PMMA (19% vs. 0%, *p* = 0.05), underscoring the need for material optimization in load-bearing applications [21]. Surface quality deficiencies manifest as suboptimal texture ratings and marginal inflammation (79% “bravo” ratings), potentially increasing biofilm accumulation and necessitating meticulous hygiene protocols [201]. The predominantly short-term clinical studies (<12 months) leave critical questions regarding long-term color stability, wear performance, and structural integrity under extended functional loading unresolved. Although 3D-printed provisionals demonstrate promising mechanical properties (flexural moduli of 2.4–2.6 GPa), these values remain significantly below natural dentin (18–25 GPa), potentially affecting stress distribution at tooth–restoration interfaces [62]. Moreover, the multi-step digital workflow introduces numerous potential error points in scanning, design, printing, and post-processing that can compromise restoration integrity, while the substantial initial investment and ongoing material costs may limit widespread adoption, particularly in resource-constrained clinical settings.

## 5. Conclusions and Future Perspectives

The integration of 3D printing technology in the fabrication of provisional crowns and bridges has demonstrated significant advancements in dental practice, particularly in terms of efficiency, customization, and material performance [25,28,29,205]. The utilization of intraoral scanning combined with digital light processing (DLP) and stereolithography (SLA) enables the production of highly accurate and esthetically pleasing restorations using biocompatible, tooth-colored resins [179,182]. These 3D-printed restorations exhibit superior or comparable mechanical properties, including enhanced fracture strength, flexural strength, elastic modulus, peak stress, and wear resistance, positioning them as durable alternatives to traditional and CAD/CAM milled methods [21,25].

The advantages of 3D printing extend beyond mechanical performance; the technology facilitates precise customization tailored to individual patient needs, significantly reducing fabrication time—from over 20 min for conventional methods to just over 5 minutes for 3D-printed crowns [25]. This digital workflow not only enhances treatment efficiency but also ensures improved marginal adaptation and internal fit, contributing to better clinical outcomes and patient satisfaction [21,131].

Despite these advancements, challenges remain, particularly concerning the long-term performance of 3D-printed materials. Issues such as color stability, water sorption, and solubility need to be addressed to enhance the effectiveness of these materials in clinical settings [18,19]. Additionally, the potential reproductive toxicity of 3D print resins warrants careful consideration [176]. Future research should prioritize optimizing resin formulations, improving filler–matrix adhesion, and exploring innovative chemistries to bolster mechanical strength and biocompatibility. Additionally, the integration of emerging technologies, such as Temperature Controlled Mask Image Projection Stereolithography (TCMIP-SL) and Digital Immediate Tooth Restoration (DITR), holds promises for further enhancing precision and efficiency in restorative dentistry [206,207,208]. Moreover, the strategic integration of AI is driving advancements in 3D printing technology, enhancing both software applications, such as design optimization and nesting, and hardware manufacturing processes. By analyzing tooth anatomy in detail and recommending suitable materials, AI enables the production of precise crowns, bridges, and dentures. This progress offers faster, more cost-effective, and clinically reliable solutions for patients [209,210,211]. On the other hand, the potential of 4D printing, with its ability to incorporate smart, stimuli-responsive materials, presents an exciting future avenue. These materials could enable resin-based provisional crowns and bridges to adapt dynamically to intraoral conditions such as temperature changes or occlusal forces, improving fit, functionality, and patient comfort. While still in its nascent stages, this technology holds the promise of redefining interim dental restorations by introducing adaptive and self-adjusting capabilities [14,212,213].

Furthermore, the highly cross-linked architecture of 3D-printed resins makes them essentially non-biodegradable, with few viable recycling routes. Predominant disposal methods—landfill and incineration—contribute to environmental pollution and the potential leaching of toxic additives (e.g., light stabilizers). Future efforts should focus on developing bio-based or recyclable resin chemistries and refining post-processing protocols to reduce solvent and energy consumption.

In conclusion, while 3D printing in dentistry presents substantial opportunities for improving restorative practices, ongoing research and development are crucial to overcoming existing limitations and expanding the clinical applications of these innovative materials. The future of digital dentistry is poised for transformative advancements that will ultimately enhance patient outcomes and redefine restorative practices.

## Figures and Tables

**Table 1:** Commercial 3D print resin for provisional crowns and bridges [38,44,45,61].

Name	Matrix	**Inorganic Fillers**
Detax FreePrint Temp (detax, Ettlingen, Germany)	Isopropylidenediphenol Peg-2 Dimethacrylat (45-<60 wt%); HEMA (1-<5 wt%); TPO (1-<5 wt%); HPMA (1-<5 wt%); Phenyl-bis(2,4,6-trimethylbenzoyl)-phosphinoxid (<1 wt%)	Not specified
DWS Temporis (DWS, Thiene, Italy)	Not specified	Not specified
Envisiontec E-dent 100 (DeltaMed GmbH, Friedberg, Germany)	Methacrylates	May include inorganic or organic fillers
Envisiontec E-dent 400 (DeltaMed GmbH, Friedberg, Germany)	Methacrylates	Contains inorganic or organic fillers
NextDent (vertex dental) C&B (Vertex-Dental B.V., Soesterberg, The Netherlands)	Not specified	Inorganic fillers
NextDent (vertex dental) C&B MFH (Vertex-Dental B.V., Soesterberg, The Netherlands)	7,7,9 (or 7,9,9)-trimethyl-4,13-dioxo-3,14-dioxa-5,12-diazahexadecane-1,16-diyl bismethacrylate; ethylene dimethacrylate; HEMA; TPO; E-BPA; mequinol; 4-methoxyphenol; hydroquinone monomethyl ether	Silicon dioxide
Stratasys VeroGlaze, MED620 (Stratasys, Minnetonka, MN, USA)	Acrylates	May include organic or inorganic fillers
3Delta temp (Deltamed, Friedberg, Germany)	Methacrylates	Silicon dioxide; dental glass (30% vol)
GC temp Print (GC America Inc., Alsip, IL, USA)	UDMA(50-<75%), TEGDMA (10-<25%); 4,4′-isopropylidenediphenol, ethoxylated and 2-methylprop-2-enoic acid (2.5-<5%); TPO (1-<2.5%); 2-(2 H-benzotriazol-2-yl)-p-cresol (0.1 < 0.2%)	Quartz 10-<25% vol
Grandio disc (Voco, Cuxhaven, Germany)	UDMA+ DMA (14%)	Nanohybrid fillers (86% *w*/*w*)
Luxatemp (DMG, Hamburg, Germany)	UDMA; Aromatic dimethacrylate; Glycol methacrylate	Glass; silica filler
CLEAR FLGP04 (Formlabs, MA, USA)	Methacrylate oligomer (75–95%); methacrylate monomer (25–50%); and diphenyl oxide (2,4,6-trimethylbenzoyl) phosphine (<1%)	Not specified
Everes Temporary (Sisma, Vicenza, Italy)	Aliphatic difunctional methacrylate (<50%); 2.2-ethylenedioxydiethyl dimethacrylate (<40%); aliphatic urethane acrylate (<20%); phosphine oxide (<2.5%); and ethylenedioxydiethyl dimethacrylate (<40%)	Not specified

**Table 2:** Summary of mechanical properties from representative reviewed studies.

Author and Year	Studied Characteristics	Evaluated Materials	Specimen Fabrication	3D Printing Parameters	Key Findings:
Mârtu et al. (2022) [71]	Surface roughness, mechanical strength	1. Superpont C + B: Heat-cured PMMA 2. Zotion PMMA: Milled PMMA 3. Freeprint Temp: Methacrylate-based resin	1. Conventional heat-curing 2. Subtractive CAD/CAM 3. Additive 3D printing	Not specified	-CAD/CAM milled PMMA exhibited highest mechanical strength -3D-printed resin showed highest initial surface roughness -All materials demonstrated significant roughness reduction post-polishing (*p* < 0.001)
Sang-Mo Park et al. (2020) [72]	Flexural strength of 3-unit fixed dental prostheses fabricated using different 3D printing technologies (DLP, SLA, FDM) versus conventional and subtractive methods	1. CV: Jet Tooth Shade™ Powder 2. SM: ViPi block monocolor 3. DLP: NextDent C&B 4. SLA: Standard (GPGR04) 5. FDM: PLA -CV/SM/DLP/SLA: Polymethyl methacrylate (PMMA)-based resins	1. CV: Self-cured PMMA resin 2. SM: Milled PMMA resin disk 3–4. DLP/SLA: Printed with PMMA-based photopolymer resin, post-cured 5. FDM: Printed with PLA filament (no post-curing)	-DLP/SLA: 100 μm layer thickness, 30° build angle Washed in 100% isopropyl alcohol, post-cured (DLP: 120 min; SLA: 60 min) -FDM: 200 μm layer thickness, 30° build angle	-DLP/SLA: Significantly higher flexural strength than CV group (*p* < 0.001) -SLA: Highest flexural strength (1323 N) -FDM: No fracture (only deformation) -DLP vs. SM: No significant difference
Al-Qahtani et al. 2021 [73]	Fabrication techniques for interim dental resins: CAD/CAM, 3D printing (stereolithography), heat-activated PMMA	1. CAD/CAM: Ceramill Temp 2. 3D Printing: Freeprint Temp 3. Conventional: Jet Tooth Shade -CAD/CAM and conventional: Polymethylmethacrylate (PMMA)	1. CAD/CAM: Milled from PMMA blanks 2. 3D Printing: SLA 3. Conventional: Heat-activated PMMA poured into molds	-Not explicitly specified -3D Printing: UV light curing (5 min in 220 V chamber) after 99% isopropyl alcohol rinse	-Surface Roughness: 3D > CAD/CAM ≈ Conventional -Microhardness: 3D > CAD/CAM ≈ Conventional -Flexural Strength: CAD/CAM ≈ 3D > Conventional
Pantea et al. 2022 [62]	Mechanical behavior (compressive strength, flexural strength)	1. 3DCS: DLP NextDent C&B MFH, microfilled hybrid PMMA resin 2. 3DOS: LCD-based vat polymerization; microfilled composite PMMA-like resin 3. CAP: Duracyl 4. CHP: Superpont C + B	1–2. 3D-printed: CAD/CAM (NextDent 5100 DLP printer, Vertex B.V., Soesterberg, The Netherlands for 3DCS; Phrozen Sonic Mini 4K LCD printer, Phrozen Technology, Xiangshan Dist., Hsinchu, Taiwan for 3DOS) 3–4 Conventional: Auto-polymerized (CAP) and pressure/heat-cured (CHP) acrylic resins	-50 μm (for 3DCS) -3DCS: 30 min UV curing at 60 °C -3DOS: 30 min UV curing	-3D-printed resins showed 2× higher elastic moduli (2.4–2.6 GPa vs. 1.3 GPa conventional) -40–88% higher bending strength in 3D-printed resins -Additive manufacturing produced more homogeneous materials compared to conventional methods
Tasın & Ismatullaev et al. (2022) [74]	Mechanical properties under thermal aging	1. Auto-polymerized PMMA 2. Bis-acryl resin 3. CAD/CAM PMMA 4. 3D-printed composite resin	1. PMMA: Manual packing 2. Bis-acryl: Injection molding 3. CAD/CAM: Milling 4. 3D-printed: SLA	-Layer thickness: 60 µm -Build orientation: 90° -Laser scanning speed: 5000 mm/s -Ultraviolet polymerization unit for 30 min	1. Digitally fabricated materials (CAD/CAM/3D-printed) showed superior mechanical properties vs. conventional 2. But CAD/CAM/Milled had highest toughness and stability after thermocycling
Henderson et al. (2022) [49]	-Effects of loading rate (1 mm/min vs. 10 mm/min) -Effects of storage time (1 day vs. 30 days in 100% humidity)	1. Milled PMMA: Solid Shade Clinical PMMA Disk (TD Dental Supply) 2. 3D-printed bis-acryl: Dentca Crown and Bridge Resin: dimethacrylate-based: bis-GMA/UDMA 3. Chairside bis-acryl: 3M-Paradigm (3M Oral Care)	1. Milled: Subtractive CAD/CAM (Sirona inLab MC x5) 2. 3D-printed: DLP 3. Chairside: Autopolymerizing resin injection via PVS matrix	-3D-printed: Layer orientation not specified (anisotropic properties noted) -Post-processing: Manufacturer-dependent polymerization	-Milled PMMA: Highest initial failure load (729 ± 113 N at 1 day), reduced after 30 days -3D-printed bis-acryl: Lowest failure load (363–523 N), further reduced after 30 days -Chairside bis-acryl: Stable strength over 30 days -Milled PMMA’s strength advantage over chairside bis-acryl diminished after 30 days -3D-printed specimens showed anisotropic properties due to layer orientation
Anthony Tahayeri et al. (2018) [15]	Printing parameters (orientation, layer thickness, resin color), mechanical properties, degree of conversion	1. 3D-Printed: NextDent C&B 2. Conventional: Integrity^®^ (Acrylates, methacrylates, barium glass fillers), Jet^®^ (Methyl methacrylate (MMA))	1. 3D-Printed: SLA 2. Conventional: Silicon mold curing (same dimensions)	-Layer thickness: 25 µm, 50 µm, 100 µm -Orientation: Optimized at 90° (reduced support usage and post-processing)	-3D-printed samples: Comparable modulus to Jet^®^, higher peak stress than Jet^®^, similar to Integrity^®^ -Higher degree of conversion vs. conventional materials -Printing accuracy error: Up to 22% (width)
Simoneti et al. (2022) [75]	3D-printed (SLA/SLS) vs. conventional (acrylic/bis-acryl resin) interim crowns	1. Acrylic resin (Polymethyl methacrylate) 2. Bis-acryl resin (Dimethacrylates, nanoparticles) 3. SLS resin: Nylon 12 (PA2201) 4. SLA resin: Methacrylates, photoinitiators	1–2 Conventional: Matrix-assisted elastomeric impression technique 3–4 3D-Printed: Digital design + SLA/SLS printing	-Layer thickness: Not specified -Orientation: Printed horizontally (only protocol tested) -SLA: 60 °C for 30 min via LED curing -SLS: None (per manufacturer)	-SLS: Highest flexural strength (+30% vs. conventional) -SLA: Lowest Vickers hardness and fatigue resistance (4/10 specimens fractured) -3D-printed materials: Lower elastic moduli (~50% vs. conventional) -Surface roughness: SLA ≈ bis-acryl resin (lowest); SLS = highest -No biofilm formation differences
Ellakany et al. (2022) [76]	-CAD/CAM milling vs. 3D printing (SLA and DLP) vs. conventional PMMA. -Effects of thermo-mechanical aging on mechanical properties	1. Conventional: Unifast Trad, auto-polymerized PMMA 2. Milled: Telio CAD, Cross-linked PMMA 3. SLA ND: NextDent C&B MFH 4. DLP AS: ASIGA Denta Tooth, methacrylate resin	1. Conventional: Manual mixing and molding 2. Milled: CAD/CAM milling 3–4. 3D-Printed: SLA and DLP	-Layer thickness: 50 μm -Orientation: 90° for both SLA and DLP -SLA ND: LC-D Print Box (3D Systems) for post-curing -DLP AS: Asiga Flash UV Curing Chamber	-Milled PMMA: Highest flexural strength (174.42 MPa) and microhardness (27.13 μm) -SLA ND: Comparable to milled PMMA except in elastic modulus -DLP AS: Similar to conventional PMMA
Reeponmaha et al. (2020) [77]	Fracture strength and patterns after thermo-mechanical aging; CAD/CAM vs. conventional methods	1. Unifast Trad: Mono methacrylate resin 2. Protemp 4: Bis-acryl composite 3. Brylic Solid: PMMA 4. Freeprint Temp: Bis-acrylate resin	1–2: Conventional direct 3: CAD/CAM milling 4: CAD/CAM 3D printing	-Post-printing: External surface light-cured -5000 thermocycles (5–55 °C) +100,000 occlusal cycles (100 N)	-Unifast Trad showed lowest fracture strength (657.87 N ± 82.84) -CAD/CAM and bis-acryl groups had comparable strength (953–1125 N) -Mono methacrylates showed Class I fractures vs. Class II in bis-acryls
Alam et al. (2022) [78]	Fracture resistance of anterior provisional crowns fabricated by different techniques	1. Group I: Protemp 4 (Bis-acryl-based resin) 2. Group II: Dentsply Sirona PMMA disk 3. Group III: NextDent C&B Resin, methacrylate-based resin	Conventional indirect (Group I), CAD/CAM milling (Group II), 3D printing (Group III)	-Layer thickness: 50 μm -Vertical building orientation -Post-printing: 30 min curing in NextDent LC-3DPrint Box (350–550 nm wavelength)	-3D-printed crowns showed highest fracture resistance (1243.18 N ± 68.18) > CAD/CAM (960.84 N ± 37.49) > Conventional (558.85 N ± 22.33) -Post-polymerization with dedicated curing units significantly enhanced material properties in 3D-printed group
Martín-Ortega et al. (2022) [79]	Fracture resistance of anterior (central incisor) and posterior (premolar) screw-retained implant-supported interim crowns fabricated via subtractive (milled) and additive (DLP) manufacturing methods	1. Milled subgroup: Vivodent CAD Multi (PMMA-based interim material) 2. Additive subgroup: Photopolymer resin, SHERAprint-cb (Rapid Shape GmbH, Heimsheim, Germany), printed with SHERAprint 30 (Rapid Shape GmbH, Heimsheim, Germany)	-Milled: 5-axis milling machine -Additive: DLP 3D printing	-Layer thickness: 50 µm -Building orientation: 45° (additive subgroup) -Ultrasonic cleaning in 98% isopropyl alcohol (2 baths), followed by UV polymerization (25 min at 220 W)	-Milled specimens exhibited significantly higher fracture resistance than additive groups (anterior: 988.4 N vs. 636.5 N; posterior: 423.8 N vs. 321.3 N) -Anterior crowns outperformed posterior crowns in both methods -All fractures occurred in crowns, not abutments
Majed M. Alsarani et al. (2023) [80]	-Milled vs. 3D-printed provisional crowns -Crown thickness (1.5 mm vs. 0.9 mm) -Thermal aging (5000 cycles between 5 °C and 55 °C)	1. Milled Material: Telio CAD PMMA) 2. 3D-Printed Material: NextDent C&B MFH (Microfilled hybrid resin)	-Milling: Zenotec t1 5-axis dental milling machine -3D Printing: NextDent 5100 printer	-3D-printed layer thickness: 0.075 mm (horizontal orientation inferred but not explicitly stated)	-Milled crowns (non-aged, 1.5 mm) showed highest fracture resistance (1706.36 N) -3D-printed crowns (aged, 0.9 mm) had lowest fracture resistance (552.49 N) -Thermocycling significantly reduced strength (*p* < 0.01)
Abad-Coronel et al. (2023) [81]	Fracture resistance of CAD/CAM materials (milled-derived PMMA vs. 3D-printed resins: 3DPPa and 3DPPb)	1. PMMA: Ivoclar Vivadent 2. 3DPPa: SprintRay (Hybrid resin (ceramic-polymer composite)) 3. 3DPPb: SprintRay (Nano ceramic hybrid (ceramic-resin composite))	-PMMA: CNC milling -3DPPa/3DPPb: DLP	-Layer thickness: 50 μm -UV light curing for 9 min	-PMMA showed highest fracture resistance (1427.9 N ± 36.9), followed by 3DPPa (1231 N ± 380.1) and 3DPPb (1029.9 N ± 166.46) -All materials exceeded average masticatory forces (~720 N)
Revilla-León et al. (2021) [82]	Chemical composition, Knoop hardness, surface roughness, shear bond strength	1. CNV Group: -Protemp 4 (3M ESPE, High silica (42.99% Si)) -Anaxdent (Anaxdent) 2. AM Group: -FreePrint Temp -AM-2: E-Dent 400 C&B MFH, High Titanium (8.64%) -AM-3: NextDent C&B MFH, Mixed elements (Na, Si, K, Cl, Ti) -AM-4: Med620 VEROGlaze, Oxygen (84.66%), Sodium (3.06%), Titanium (2.05%)	CNV: Silicone index + glass tile AM: CAD-designed disks printed with 90° build orientation	-Layer thickness: 50 μm, -Building orientation: 90° for all AM groups -Post-polymerization: UV curing (6–30 min): -AM-1: 6 min -AM-2: 15 min -AM-3: 30 min -AM-4: None required	-Significant chemical variations between AM/CNV groups -AM-4 (13.45 KHN) and CNV-2 (13.35 KHN) showed highest hardness -AM-1 had highest roughness (1.88 Ra) -No significant bond strength differences
Soto-Montero et al. (2022) [83]	Effect of post-curing time (0, 5, 10, 15, 20 min) on -Flexural strength (FS) -Flexural modulus (FM) -Microhardness (KHN) at varying depths	1. Cosmos Temp3D (COS) (Methacrylate oligomers, diphenyl-2,4,6-trimethylbenzoyl phosphine oxide, TiO_2_, carbon black) 2. SmartPrint BioTemp (SMA) (Methacrylic ester monomers, stabilizers, fillers, pigments) 3. Resilab3D Temp (RES) (Undisclosed) 4. Prizma3D BioProv (PRI) Methacrylic acid esters, acrylic oligomers, pigments	3D-printed using DLP technology (Anycubic Photon printer, Anycubic Technology Co., Shenzen, China)	-50 μm printed at 0° angulation -Anycubic Wash and Cure 2.0 unit (violet LED, 390–410 nm peak at 401 nm), irradiance 4–10 mW/cm^2^, rotational curing platform	-Post-curing time significantly influenced ΔE_00_, FS, FM, and KHN -COS and SMA exceeded ΔE_00_ acceptability thresholds after 5 and 10 min, respectively -FS plateaued earlier for RES (5 min), PRI/SMA (10 min) -Longer post-curing increased surface KHN but reduced depth polymerization efficiency
Bayarsaikhan et al. (2021) [84]	Post-curing temperature (40 °C, 60 °C, 80 °C) and duration (15–120 min)	Denture Teeth Resin A2 (Formlabs, Somerville, MA, USA): PMMA-based acrylic resin with (meth)acrylate monomers (exact composition not specified)	SLA	**-Layer thickness:** 50 µm -Bars: 120° orientation, Disks: 0° orientation -FormCure UV-light chamber (405 nm, 13 LEDs) at 40–80 °C for 15–120 min; cleaned with 90% isopropanol	-Higher post-curing temperature (80 °C) and longer duration (120 min) improved flexural strength (147.48 MPa) and cell viability (89.51%) -Lower cytotoxicity and protein adsorption at 80 °C -Post-curing time > 60 min showed minimal incremental benefits
Mayer et al. (2021) [85]	Cleaning methods (Isopropanol, Yellow Magic 7, Centrifugation) and their impact on wear/fracture load; comparison of 3D-printed vs. milled PMMA FDPs	1. Freeprint Temp (FPT: Methacrylates, photoinitiators (no fillers)) 2. GC Temp PRINT (GCT: Methacrylates, quartz fillers) -C&B MFH (NMF: Unspecified resin with inorganic fillers) 3. TelioCAD (TEL: PMMA polymer (no fillers))	-3D-printed: DLP -Milled: Wet grinding process	Not specified in the provided content	-Printed FDPs showed superior wear resistance but lower fracture load vs. milled PMMA -Isopropanol cleaning reduced fracture load vs. centrifugation/Yellow Magic -Fillers improved wear resistance but not fracture load
Aldahian et al. (2021) [86]	Marginal integrity, surface roughness, wear of interim crowns	1. Conventional (CN): Jet Tooth Shade™ Self-Curing Acrylic Resin (Lang Dental Manufacturing Co. Inc.) 2. CAD/CAM (CC): Cercon base PMMA blocks (DeguDent GmbH, Hanau, Germany) 3. 3D Printing (3D): Freeprint Temp resin (DETAX GmbH & Co. KG, Ettlingen, Germany)	-CN: Manual mixing, molding, and polymerization -CC: CAD/CAM milling (Versamill machine) -3D: SLA	-Layer Thickness: 50 μm -Orientation: 0° (flat printing) -Soaked in 99% isopropyl alcohol (60 s), UV-cured (90 s in curing chamber)	-Adaptation:CN and CC showed comparable adaptation (*p* > 0.05); 3D had lowest values (197.82 ± 11.72 μm) -Marginal Misfit:CN had highest misfit (395.89 ± 80.33 μm); 3D and CC performed better (*p* < 0.05) -Surface Roughness: CC had lowest Ra (3.28 ± 0.34 μm); 3D had highest (5.61 ± 0.33 μm) -Surface Wear: 3D showed lowest wear (10.81 ± 2.00 mm^3^); CN had highest (17.79 ± 2.78 mm^3^) -Post-Treatment: Critical for 3D-printed specimens to achieve optimal mechanical properties
Park et al. (2018) [40]	Wear resistance of 3D-printed, milled, and self-cured resin materials opposing zirconia and CoCr alloy antagonists	1. C&B: NextDent 2. Vipi Block: VIPI 3. Jet™: Lang Dental Mfg -All materials: PMMA-based (Poly Methyl Methacrylate)	-3D-printed resin: DLP 3D printing (Veltz3D D1-150) -Milled resin: Dry milling (Roland DWX-51D) -Self-cured resin: Conventional pressure curing in a mold	-Layer thickness: 100 μm -Build angle: 0° (tested surface parallel to build platform) -Post-wash with 100% isopropyl alcohol -Post-curing for 120 min (Denstar-300 machine)	-No significant difference in wear resistance between 3D-printed, milled, and self-cured resins -SEM revealed cracks and interlayer bond separation in 3D-printed resin when opposing metal abraders -No significant wear difference between zirconia and metal antagonists
Kessler et al. (2019) [87]	Three-body wear resistance of temporary dental materials	1. 3Delta temp 2. NextDent C&B 3. Freeprint temp 4. Telio CAD 5. Tetric EvoCeram (Bis-GMA, UDMA, barium glass (75–76 wt% filler))	-3D-printed: DLP -Telio CAD: Milled from blocks -Tetric EvoCeram: Direct light-curing	-Layer thickness: Not explicitly stated -Parallel to build platform -ltrasonic cleaning (ethanol) -Post-curing: 2 × 2000 flashes under nitrogen (Otoflash G171)	-Lowest wear: Tetric EvoCeram (50 ± 15 µm), 3Delta temp (62 ± 4 µm) -Highest wear: Freeprint temp (257 ± 24 µm) -Filler content directly correlated with wear resistance -3Delta temp outperformed other 3D-printed materials due to higher filler content
Digholkar et al. (2016) [88]	Fabrication methods: Rapid prototyping (RP), CAD/CAM milling (CC), conventional heat-activated PMMA (CH)	1. RP: Envision TEC E-Dent 100 (Light-cured micro-hybrid filled composite) 2. CAD/CAM: Ceramill TEMP (PMMA) 3. Conventional: Heat-activated PMMA (GC Corporation)	-RP: 3D printing with UV light polymerization -CAD/CAM: Milling of pre-polymerized blanks -Conventional: Compression molding	-Layer-by-layer additive manufacturing (vertical stacking) -UV oven curing for RP specimens	-Highest flexural strength: CAD/CAM (104.20 MPa) -Highest microhardness: RP (32.77 KHN) -No significant difference between CAD/CAM and conventional flexural strength (*p* = 0.64)
Reymus et al. (2020) [89]	-Build direction (occlusal, buccal, distal) -Post-curing methods -Artificial aging (H_2_O immersion for 21 days at 37 °C)	1. 3D-printed resins: Experimental (EXP, GC Europe), NextDent C&B (CB, NextDent), Freeprint temp (FT, Detax), 3Delta temp (DT, Deltamed) -Methylmethacrylate for all 3D-printed materials 2. Controls: TelioCAD (TC, Ivoclar-Vivadent)	-3D-printed using SLA/DLP technology (D20II printer, Rapid shape, Heimsheim, Germany) -Positive control (TC) milled from PMMA blocks -Negative control (LT) fabricated via conventional interim material technique	-Long-axis orientations: occlusal, buccal, or distal relative to the printer’s platform -Labolight DUO (6 min LED curing) -Otoflash G171 (4000 flashes -LC-3DPrint Box (30 min UV curing)	-Distal build direction yielded higher fracture load vs. occlusal -CB showed highest fracture load, EXP the lowest -Post-curing strategy significantly impacted mechanical stability -EXP and DT exhibited reduced fracture load after artificial aging
Schulz et al. (2023) [42]	Interim resin (VarseoSmile Temp A2) vs. permanent ceramic-filled hybrid resin (VarseoSmile Crown Plus A2)	1. Interim resin: Methacrylic acid esters, diphenyl phosphine oxide 2. Permanent resin: Methacrylic acid esters, silanized dental glass, inorganic fillers (30–50% by mass)	3D printing via DLP	Not explicitly stated in the paper (marked N/A) -Ultrasonic cleaning in ethanol (96%) -Nitrogen gas (1.0–1.2 bar) + otoflash device (1500 flashes, 10 Hz)	-No significant difference in fracture resistance: 365.90 ± 86.67 N (interim) vs. 363.45 ± 87.57 N (permanent) -Both materials exhibited sufficient resistance to masticatory forces
Alshamrani et al. (2023) [45]	Mechanical properties (flexural strength), biocompatibility (cell viability)	1. Resin: B2 Everes Temporary (Sisma, Vicenza, Italy) 2. Nanoparticles: Glass silica (GM35429) and zirconia (GM018-307) (Schott, Landshut, Germany)	3D-printed using DLP	Layer Thickness: Not explicitly stated in the provided content; 90-degree orientation from the build platform	-5% glass silica and 10–20% zirconia significantly improved flexural strength (113.8–114.6 MPa) -All groups showed >80% cell viability (non-cytotoxic) -Higher zirconia concentrations enhanced biocompatibility (>94%)
KEßLER et al. (2021) [90]	Printing direction (horizontal parallel, horizontal perpendicular, vertical), aging (thermocycling: 10,000 cycles at 5–55 °C)	1. NextDent C&B 2. 3Delta temp 3. Freeprint temp	DLP	-Layer Thickness: 1. NextDent C&B: 50 μm 2. Freeprint temp: 50 μm 3. 3Delta temp: 100 μm -Layer Orientation: 1. Horizontal parallel (force perpendicular to layers) 2. Horizontal perpendicular (force parallel to layers) 3. Vertical (force perpendicular to layers) -Post-curing: 1. NextDent C&B: LC-3D Print Box (30 min) 2. Freeprint temp and 3Delta temp: Otoflash G171 (2 × 2000 flashes under nitrogen)	1. Printing direction significantly affected FS/FM (η_p_^2^ = 0.407 for FS in 3Delta temp) 2. Aging reduced FS by 12–25% 3. 3Delta temp (highest filler content) outperformed others in FS and FM
Eun-Hyuk Lee et al. (2022) [91]	Layer thickness (25 µm, 100 µm), printing orientation (0°, 45°, 90°)	C&B 5.0 Hybrid (ARUM)	DLP 3D printing, post-cleaned with isopropyl alcohol, UV-polymerized, ground with abrasive paper	-Layer thickness: 25 and 100 µm -Layer Orientation: 0°, 45°, 90° -Post-curing: UV light polymerization (PURE PRO unit)	-Thinner layers (25 µm) showed higher discoloration -0° orientation had better color stability vs. 45°/90° -Red wine caused continuous discoloration; coffee peaked at 15 days -Surface roughness and contact angle unaffected by parameters
Scherer et al. (2023) [92]	Layer thickness (10–150 μm) and artificial aging (thermocycling) effects	NextDent C&B MFH (3D Systems)	DLP	-Layer thickness: 10, 25, 50 (control), 75, 100, 125, 150 μm -Orientation: Perpendicular to load direction -Post-curing: 30 min in LC-3DPrint Box (300–550 nm light), after 3 + 2 min IPA rinsing	-Layer thickness did not significantly affect flexural strength -Artificial aging reduced flexural strength (80.33% variance explained) -Highest Weibull modulus for nonaged 50/75 μm and aged 125 μm specimens
Almira Ada Diken Turksayar et al. (2022) [93]	Fracture strength of 3-unit interim FDPs under different printing orientations (0°, 30°, 45°, 90°, 150°) and manufacturing methods	1. Additive: Temporary CB (Methacrylate-based resin) 2. Subtractive: DuoCAD (PMMA, FSM Dental)	-Additive: SLA -Subtractive: Milled with CEREC MC X5	-Layer thickness: 50 μm for SLA printing -Orientation: 0°, 30°, 45°, 90°, 150° relative to the build platform -Post-curing: -Washed in isopropyl alcohol -Cured twice (20 min each, 60 °C) in Form Cure unit	-0° and 30° printed FDPs showed fracture strength comparable to milled PMMA -90° orientation had the lowest strength -Milled FDPs outperformed 45° and 150° printed FDPs
Derban et al. (2021) [43]	Printing angle (0°, 45°, 90°) and load direction (parallel vs. perpendicular)	1. NextDent MFH Vertex Dental (Vertex Dental B.V, Soesterberg, The Netherlands) 2. Detax Freeprint Temp (Detax GmbH & Co KG, Ettlingen, Germany)	SLA	-Layer thickness: 50 microns -Orientation: 0°, 45°, 90° (additional 0° specimens for load direction testing) -Post-curing: -Ultrasonic cleaning in ethyl alcohol (3 cycles) -Nitrogen gas curing in Otoflash unit	-Printing angle significantly affects flexural strength (*p* < 0.001) -0° and 90° orientations showed better mechanical performance than 45° -Parallel loading direction to layer growth increased strength.
Unkovskiy et al. (2018) [94]	Build orientation (0°, 45°, 90°), positioning on build platform, curing methods	Dental SG: Photopolymer resin (class I biocompatible, ISO 10993-1 [95])		-Layer thickness: 50 µm -Orientation: 0°, 45°, 90° -Post-curing: 15 min curing in three units: Dentacolor XS, Speed Laborlight, Visio Beta Vario (320–520 nm wavelength) -Uncured “green state” specimens as control	-45° orientation showed highest dimensional accuracy -90° orientation had superior flexural strength/modulus -Edge positioning caused higher inaccuracies -Curing units had no significant impact on accuracy or mechanical properties -Final polymerization improved flexural properties but did not affect dimensions
Chen et al. (2023) [96]	Post-polymerization temperature (RT, 40 °C, 60 °C, 80 °C) and time (0–120 min)	AA Temp (Methacrylate-based resin (photopolymerizable))	3D-printed via DLP, cleaned with 95% alcohol	-Layer thickness: 50 μm; orientation not specified -Post-curing: FormCure unit (405 nm, 40–80 °C, 15–120 min)	-Optimal flexural properties at 60 °C/90 min -Curing depth: 3–4 mm with heating -Surface hardness improved with post-polymerization -60 °C showed superior mechanical properties compared to other temperatures -Post-polymerization without heating resulted in shallow curing depth (<1–2 mm)
Crispim et al. (2024) [97]	Build direction (30° vs. 90°), post-polymerization time (15, 30, 45 min), aging (90-day water storage at 37 °C)	Provisional resin: Cosmos TEMP	3D-printed provisional crowns	-Layer thickness: 50 μm. -Orientation: 30° and 90° relative to the horizontal plane -UV post-curing (405 nm, 40 W) with Anycubic Wash & Cure Plus (Anycubic Technology Co., Shenzen, China), durations: 15, 30, 45 min	-30° build direction improved marginal/internal adaptation, fracture resistance, and margin quality -Post-polymerization for 30 min maximized fracture resistance -Aging (90 days) reduced strength but enhanced degree of conversion -Lower MD/ID in aged 30° groups versus 90° -98.6% of 30° crowns had defect-free margins vs. 90° group with higher irregularities
Britto et al. (2022) [39]	Flexural strength (σ_f_), elastic modulus (E), water sorption (W_sp_), solubility (W_sl_), biocompatibility	1. 3D: Oligomers, monomers, photoinitiators, stabilizer, pigments 2. BA: Bis-acryl composite resin 3. AR: Heat-cured acrylic resin (PMMA-based)	1. 3D: Varseo 3D printer (Bego, Bremen, Germany) 2. BA: Silicone molds from 3D prints 3. AR: Traditional flasking/heat curing	-Layer thickness: 0.03 mm -Orientation: Horizontal -Resolution: 25 μm -LED light curing (400–450 nm, 13 min)	-3D showed increased σ_f_ vs. AR after aging -BA had lowest σ_f_/E values -All materials met ISO 10477 [98] for W_sp_/W_sl_ -AR showed reduced cell viability (71.9% vs. 92.9% for 3D) in SRB test

**Table 3:** A summary of color stability in the studies included in the review.

Author and Year	Studied Property	Evaluated Materials	Fabrication Technique	3D Printing Parameters	Key Results
Cristina Espinar et al. (2023) [1]	Color difference (Δ*E*_00_) and relative translucency parameter (*RTP*_00_)	-DFT: Detax Freeprint Temp (A1,A2,A3, GmbH) -FP: Formlabs Permanent Crown (A2,A3,B1,C2, Formlabs Inc., Somerville, MA, USA) -FT: Formlabs Temporary CB (A2,A3,B1,C2) -GCT: GC TempPrint (Light/Medium, GC Corporation, Tokyo, Japan)	-DFT and GCT: DLP (Asiga Max UV) -FP and FT: SLA (Form 3B+)	-Layer thickness: 50 µm -Orientation: 0° and 90°	-Printing orientation caused perceptible Δ*E*_00_ (>0.81) for DFT-A1/A2/A3, FP-B1/C2, FT-A2/B1 -DFT-A1 showed highest ΔE_00_ (2.94) exceeding acceptability threshold -Significant RTP changes in DFT-A1/A3, FP-B1, FT-B1 (>0.62 perceptibility threshold) -GC TempPrint showed minimal color/translucency variations -Color changes were primarily driven by ΔL* (lightness) or ΔC* (chroma), except GCT-M (hue) -Translucency Direction: Depended on material/shade (e.g., DFT increased at 90°, GCT decreased at 90°)
Soto-Montero et al. (2022) [83]	Color change (Δ*E*_00_)	1. Cosmos Temp3D (COS) 2. SmartPrint BioTemp (SMA) 3. Resilab3D Temp (RES) 4. Prizma3D BioProv (PRI)	DLP	-Layer thickness: 50 μm layer thickness -Orientation: 0°	-Post-curing time significantly influenced Δ*E*_00_, FS, FM, and KHN -COS and SMA exceeded ΔE_00_ acceptability thresholds after 5 and 10 min, respectively
Lee et al. (2022) [91]	Color stability (Δ*E*_00_) and stainability under aging media	C&B 5.0 Hybrid resin (ARUM), ASIGA MAX UV 3D printer (ASIGA Sydney, Australian): Methacrylate-based photopolymer resin (exact composition not specified)	DLP	-Layer thickness: 25 µm, 100 µm -Orientation: 0°, 45°, 90°	-25 µm layers showed higher Δ*E*_00_ than 100 µm -0° orientation had lowest discoloration -Coffee caused initial ΔE_00_ increase then decrease; wine caused continuous increase
Al-Akhali et al. (2023) [131]	Color stability (ΔE*) after 1 and 7 days of immersion in khat extract vs. distilled water	-PMMA CAD/CAM milling: Poly-methyl methacrylate -3D Printing: Liquid resin (unspecified polymer) -PMMA SC acrylic resin: Self-cured PMMA -LC composite: Light-cured composite -Bis-acrylic SC composite: Bis-acrylate resin	-Manual: Metallic mold (PMMA SC, LC composite, Bis-acrylic) -CAD/CAM: Milled from PMMA blocks -3D Printing: DLP	-Layer thickness: 50 μm -Orientation: Not specified	-3D printing: Significant color increase over time (ΔE* = 1.96 at 1 day; 10.67 at 7 days) -Khat extract caused greater discoloration than distilled water (*p* < 0.05) -3D printing limitations: Surface roughness and lower polymerization rates contributed to higher color instability
Yıldırım et al. (2024) [128]	Aging resistance after thermocycling	1. PEMA: Dentalon Plus 2. BRC: Prevision Temp 3. Milled: Tempo CAD 4. Printed: Cura Temp	Not specified	-Layer thickness: layer thickness 100 μm -Orientation: perpendicular (90°) orientation to build platform	-Highest Δ*E*_00_ in BRC (3.2) and printed (3.0) -Lowest Δ*E*_00_ in PEMA (1.1) and milled (1.3) -Printed/BRC exceeded clinical acceptability threshold (ΔE_00_ > 1.8)
Alalawi et al. (2024) [129]	Color stability	1. UNIFAST III: Methyl methacrylate 2. NextDent C&B MFH: Methacrylic oligomers + phosphine oxides 3. FormLabs Temporary CB Resin: Biocompatible photopolymer resin 4. TelioCAD: 99.5% PMMA + 0.5% pigment	1. Cold-cured mold 2–3. 3D-printed 4. CAD/CAM milled	Not specified	-Coffee/tea caused greatest ΔE (3.22–19.03 NBS units), especially on unpolished surfaces -3D-printed materials exhibited greater susceptibility to dietary solvents compared to milled -Surface finishing significantly impacted results (unpolished surfaces more vulnerable)
Almejrad et al. (2021) [132]	Color stability (Δ*E*, ΔL*) using CIE Lab* system	NextDent Crown & Bridge A3.5: Methacrylate-based photopolymerizing resin	3D-printed full-coverage interim crowns	Not specified	-Highest Δ*E*: Wine (24.92 ± 4.6 for polish, 19.17 ± 2.5 for Optiglaze) -Optiglaze reduced coffee-induced discoloration (ΔE 2.82 vs. 7.89 in polish) -All groups exceeded ΔE acceptability threshold (2.7)

**Table 4:** Summary of water sorption and solubility from representative reviewed studies.

Author and Year	Evaluated Materials	Shape and Dimension of Tested Resins Samples	Result of Water Sorption (Wsp)	Result of Water Solubility (Wsl)
Britto et al. (2022) [39]	1. 3D-printed polymer (Cosmos Temp) 2. Bis-acryl composite resin (Yprov Bis-acryl) 3. Heat-cured acrylic resin (Coroas e Pontes)	Bar-shaped specimens: -Length: 25 ± 2 mm -Height: 2 ± 0.1 mm -Width: 2 ± 0.1 mm Disk-shaped specimens: -Diameter: 15 ± 1 mm -Thickness: 1 ± 0.1 mm	-3D-printed polymer: 14.4 μg/mm^3^ -Lowest water sorption among tested materials -Well below ISO 10477:2020 limit of 40 μg/mm^3^	-3D-printed polymer: 3.5 μg/mm^3^ -Shared value with Bis-acryl resin -Significantly lower than the ISO 10477:2020 limit of 7.5 μg/mm^3^
Leila Perea-Lowery et al., 2021 [140]	1. Imprimo^®^ LC Denture (Scheu-Dental GmbH, Iserlohn, Germany) 2. Paladon^®^ 65 (Kulzer GmbH, Mitsui Chemicals, Hanau, Germany) 3. Palapress^®^ (Kulzer GmbH, Mitsui Chemicals, Hanau, Germany)	Bar-shaped specimens 10.0 × 65.0 × 3.3 ± 0.2 mm^3^	Mean values: -3D-printed Imprimo Cure^®^: 2.2 ± 0.01% -3D-printed Form Cure^®^: 2.2 ± 0.008%	Mean values: -3D-printed Imprimo Cure^®^: 0.67 ± 0.024% -3D-printed Form Cure^®^: 0.55 ± 0.027% -Paladon^®^: 0.32 ± 0.024% (lowest)
Veronika Greil et al., 2023 [139]	1. NextDent Denture 3D+ (DEN)—NextDent, Soesterberg, Netherlands 2. Fotodent Denture (FOT)—Dreve ProDiMed, Unna, Germany 3. Freeprint Denture (FRE)—DETAX, Ettlingen, Germany 4. V-Print dentbase (VPR)—VOCO, Cuxhaven, Germany 5. Ivotion Base (IVO)—Ivoclar Vivadent, Schaan, Liechtenstein 6. PalaXpress (PAL)—Kulzer, Hanau, Germany	Rectangular specimens in three geometries: -5 × 10 × 25 mm (for degree of conversion) -Specific dimensions for water sorption, flexural strength tests	-3D-printed resins: 25.31–37.94 μg/mm^3^ -Exceeds ISO norm (32 μg/mm^3^) for most materials -Highest: VPR and DEN -Lowest: FOT	-3D-printed resins: 0.08–8.27 μg/mm^3^ -Exceeds ISO norm (8 μg/mm^3^) for most materials -Highest initial values: VPR -Lowest initial values: FOT
Jain et al., 2022 [17]	Not specified in detail in the paper	Not specifically detailed in the review	-3D-printed PMMA: Higher than conventional polycarbonate, lower than conventional PMMA -3D-printed photopolymer: Higher than CAD/CAM milled PMMA and conventional bis-acrylic	-3D-printed PMMA: Higher than conventional polycarbonate and PMMA resins -3D-printed photopolymer: Higher than conventional PMMA, bis-acrylic, and CAD/CAM milled PMMA

**Table 7:** Summary of biocompatibility from representative reviewed studies.

Author and Year	Evaluated Materials	Specimen Fabrication Technique	Key Results
Atria et al. (2022) [181]	-Crowntec (CT) -Temporary C&B (FL) -C&B MFH (ND) -Permanent Bridge (PB)	3D printing followed by post-processing steps (washing, UV curing, etc.)	No significant differences in cell viability and proliferation among materials; cytotoxicity was low for all materials, with ND showing the highest LDH activity (13.7%)
Britto et al. (2022) [39]	-3D: Cosmos Temp -BA: Yprov Bis-acryl -AR: Coroas e Pontes	-3D: 3D printing (Varseo 3D printer, Bego, Bremen, Germany) with 25 μm resolution and 20–40 mm/h building rate -BA: Laboratory condensation silicone matrices -AR: Heat-cured acrylic resin	-3D structures showed higher cell viability (92.9%) compared to AR (71.9%) in SRB test -No significant difference in MTT assay (*p* > 0.05)
Folwaczny et al. 2023 [61]	-NextDent MFH -3Delta temp -GC Temp -Freeprint temp -Grandio disc -Luxatemp	-3D-printed materials: Additive manufacturing (NextDent MFH, 3Delta temp, GC Temp, Freeprint temp) -Subtractive material: Grandio disc (Voco) -Conventional material: Luxatemp (DMG)	-Luxatemp and 3Delta temp caused significant reduction in cell viability (<10% of control) and strong inhibition of IL-6 and IL-8 expression -Grandio disc and other 3D-printed materials (MFH, GC Temp, Freeprint) showed higher cell viability (>70% of control) and moderate cytokine expression
Alshamrani, A. et al. 2023 [45]	-B2 Everes Temporary (dental resin)—Sisma, Italy; Glass fillers (GM35429)—Schott, Landshut, Germany; Zirconia glass (GM018-307)—Shofu Inc., Ratingen, Germany	DLP 3D printing	Cell viability > 80% for all groups, indicating non-toxicity and biocompatibility
Wuersching et al. [175]	Permanent FDP: -Tetric EvoCeram -Tetric CAD -VarseoSmile Crown plus -NextDent C&B MFH Temporary FDP: -Protemp 4 -Telio CAD -VarseoSmile Temp -Temp PRINT -P Pro Crown & Bridge	-Additive (DLP 3D printing): Varseo XS/BEGO, P30/RapidShape, NextDent 5100 -Subtractive: Milled resin blocks (Tetric CAD, Telio CAD) -Conventional: Light-cured (Tetric EvoCeram)/self-cured (Protemp 4)	-Tetric CAD/Telio CAD (subtractive resins): Slight toxicity -All other resins: Moderate–severe cytotoxicity -VarseoSmile Crown+/P Pro Crown & Bridge: Significantly elevated PGE2 -Telio CAD/VarseoSmile Temp/P Pro: Increased oxidative stress (↑GSSG) -All printable resins: Induced slight apoptosis
Rogers, H.B. et al. (2021) [176]	-Dental SG (DSG) and Dental LT Clear (DLT), manufactured by Formlabs.	3D printing using SLA technology—specimens were post-processed with ethanol washes and UV curing	-DLT resin leachates, particularly Tinuvin 292, caused severe oocyte toxicity and chromosomal misalignment. -Plasma-treated DSG reduced oocyte degeneration but caused chromosomal abnormalities

**Table 8:** Summary of antibacterial properties from representative reviewed studies.

Author and Year	Evaluated Materials	Main Chemical Composition	3D Printing Technique	3D Printing Parameters	Key Results
Liu Sa et al. (2019) [184]	No specific trade name mentioned	-Resin components: UDMA (60 wt%), TEGDMA (40 wt%), TPO (1 wt%), -Antibacterial agent: Ag-HNT (1%, 2%, 3%)	DLP	1. Z-resolution: 50 µm 2. Layer exposure time: 12 s 3. Additional UV curing for 0.5 h after printing	-The composite resin demonstrated sustained antibacterial activity, maintaining 99% efficiency even after repeated extraction for 7 days
ElMalah et al. (2024) [189]	VarseoSmile Crown plus; BEGO	-Antibacterial agent: TiO_2_ NPs and silanized chitosan nanoparticles (sCS NPs)	DLP	1. Resolution: 25 µm 2. Layer thickness: 0.03 mm 3. Post-polymerized in a high-performance unit at 200 W for 15 min	-TiO_2_ NPs showed the highest antibacterial activity against *Streptococcus mutans*
Ye-Hyeon Jo et al. (2024) [190]	DentaBASE (Asiga, Sydney, Australia)	-Antibacterial agent: Phytochemical microcapsules (MPs) derived from phytoncide oil: (−)-α-pinene, (+)-3-carene, para-cymene	DLP	1. Layer thickness: 50 μm 2. Build orientation: 0° horizontally positioned 3. UV-based polymerization system with a light-emitting diode (LED) power output of 200 W for 200 s	-MPs reduced colony counts (*p* ≤ 0.001) and biofilm formation (*p* ≤ 0.009) for all tested species; (−)-α-pinene suppressed microbial growth effectively without cytotoxicity (*p* = 0.310)
Li et al. (2019) [192]	No specific trade name mentioned	-Resin components: TMPTA3EO (acrylate), TTT (ene), PETMP (thiol) -Antibacterial agent: QAC/SH-QAC	DLP	1. Print time: 10 min 2. Ethanol wash (1–2 min) + 405 nm LED post-curing (3–5 min)	-4 wt% QAC: >99.99% efficacy against *E. coli* and *S. aureus* -10 wt% SH-QAC: 100% efficacy against *S. aureus* -First thiol-ene-acrylate ternary system for DLP with 8.67% shrinkage (vs. >10% in conventional acrylates) -Maintained >85% double-bond conversion rate despite antibacterial additives
Wu et al. (2024) [193]	No specific trade name mentioned	-Resin components: ACMO, IBOMA, 3,3,5-trimethylcyclohexyl acrylate, bifunctional urethane acrylate resins, TPO photoinitiator -Antibacterial agent: IPDI-HEMA-choline chloride copolymer (N3)	DLP	1. Post-curing: 2 h in UV light box	-10% N3 concentration achieved >99% antibacterial rate against *E. coli*/*S. aureus* -Maintained >99% efficacy after 35 days’ storage
Gan Jin et al. (2024) [194]	-Synthesized UA resin with 0 wt% (0F-UA), 5 wt% (5F-UA), and 10 wt% (10F-UA) fluoride complex -Reference material: DENTCA Denture Base II (Torrance, CA, USA)	-Base resin: Urethane-acrylate (ethoxylated pentaerythritol tetraacrylate, aliphatic urethane hexa-acrylate, methyl methacrylate, isobornyl acrylate) -Antibacterial agent: Zirconium(IV) fluoride complex	DLP	1. Post-washing: 10 min in tripropylene glycol methyl ether 2. Post-curing: 20 min in Form cure (Formlabs)	-10F-UA showed strongest inhibition of *S. mutans* growth (direct contact: 9.96% viability; indirect: 48.75% viability) -Fluoride release correlated with antibacterial efficacy
